# A Multistate Toggle Switch Defines Fungal Cell Fates and Is Regulated by Synergistic Genetic Cues

**DOI:** 10.1371/journal.pgen.1006353

**Published:** 2016-10-06

**Authors:** Matthew Z. Anderson, Allison M. Porman, Na Wang, Eugenio Mancera, Denis Huang, Christina A. Cuomo, Richard J. Bennett

**Affiliations:** 1 Department of Microbiology and Immunology, Brown University, Providence, Rhode Island, United States of America; 2 Department of Microbiology and Immunology, University of California San Francisco, San Francisco, California, United States of America; 3 Broad Institute of MIT and Harvard, Cambridge, Massachusetts, United States of America; University of Melbourne, AUSTRALIA

## Abstract

Heritable epigenetic changes underlie the ability of cells to differentiate into distinct cell types. Here, we demonstrate that the fungal pathogen *Candida tropicalis* exhibits multipotency, undergoing stochastic and reversible switching between three cellular states. The three cell states exhibit unique cellular morphologies, growth rates, and global gene expression profiles. Genetic analysis identified six transcription factors that play key roles in regulating cell differentiation. In particular, we show that forced expression of Wor1 or Efg1 transcription factors can be used to manipulate transitions between all three cell states. A model for tristability is proposed in which Wor1 and Efg1 are self-activating but mutually antagonistic transcription factors, thereby forming a symmetrical self-activating toggle switch. We explicitly test this model and show that ectopic expression of *WOR1* can induce white-to-hybrid-to-opaque switching, whereas ectopic expression of *EFG1* drives switching in the opposite direction, from opaque-to-hybrid-to-white cell states. We also address the stability of induced cell states and demonstrate that stable differentiation events require ectopic gene expression in combination with chromatin-based cues. These studies therefore experimentally test a model of multistate stability and demonstrate that transcriptional circuits act synergistically with chromatin-based changes to drive cell state transitions. We also establish close mechanistic parallels between phenotypic switching in unicellular fungi and cell fate decisions during stem cell reprogramming.

## Author Summary

Formation of different cell types accompanies development in multicellular organisms and increases population-level diversity in unicellular species. In both instances, binary cell fate decisions are often determined by toggle switches, in which two master regulators compete to define one of two possible cell states. However, much less is known about multistate transitions due to the lack of experimentally tractable systems. Here, we demonstrate that the fungal pathogen *Candida tropicalis* undergoes reversible and heritable transitions between three stable cell states. We propose that tristability is achieved by a symmetric self-activating toggle switch and test this model by ectopic expression of the two master transcription factors. Significantly, manipulating the expression of the two master regulators induced all six possible state-to-state transitions in the tristable system. Furthermore, we found that stable inheritance of transcriptionally induced states only occurred if combined with inhibition of a histone deacetylase activity. Together, these results define the genetic regulation of a tristable switch and demonstrate that synergistic genetic cues (one transcriptional and one chromatin-based) drive epigenetic inheritance in a unicellular species. We also discuss striking parallels between cell fate decisions in yeast and the regulation of cell differentiation events in higher eukaryotes.

## Introduction

Epigenetic transitions are responsible for the ability of cells to undergo heritable changes in cell type without an underlying change in the primary DNA sequence. Such transitions accompany development in multicellular organisms, as well as the reprogramming of differentiated somatic cells into pluripotent stem cells [1, 2]. Genetic regulation of cell fates is determined by transcription factors that act in inter-connected circuits to drive lineage-specific gene expression [3–5]. Chromatin-based cues also play key roles in epigenetic inheritance, including post-translational histone modifications and remodeling of chromatin structure [6, 7].

Much of the current understanding of cell fate determination has come from analyzing differentiation events in multicellular species. Here, stable cell states have been envisaged as “valleys” in an epigenetic landscape [8, 9]. During development, cells traverse a series of bifurcation events (forks in the valleys) as they progress from pluripotency to differentiated cell types [8, 10–12]. The transcriptional regulation of bifurcation points has been investigated in detail, including the roles of PU.1/GATA1 in myeloid differentiation [10, 13], Oct4/Cdx2 in formation of the trophectoderm [14], and Oct4/Sox2 in differentiation of the mesoendoderm or neuroectoderm [15]. In these examples, mutual inhibition (MI) between lineage-specific transcription factors plays a central role in directing differentiation. MI circuits produce bistable toggle switches, and cell fate is determined by which of two alternative transcriptional programs dominates.

More recently, studies have examined cell fate choices in systems with multiple stable states. Modeling reveals that one or both transcription factors in a MI circuit must exhibit self-activation, in addition to mutual antagonism, to support multistate stability [16, 17]. One potential outcome of mutual activation/mutual inhibition (MAMI) circuits is tristability, with two cell states represented by high expression of one transcription factor or the other, and a third hybrid state formed at intermediate levels of both transcription factors [18–21]. However, experimental manipulation of multi-state systems has been limited, and analysis has often relied on transcriptional measurements and cell state modeling.

Microbial populations can also display epigenetic regulation of cell states. This can have beneficial outcomes as phenotypic variation in a unicellular population promotes “bet hedging” and enables faster adaptation to fluctuating environments [22–24]. Moreover, bacterial and fungal cells often differentiate into structured communities, producing subpopulations of phenotypically distinct cells that can coordinate their cellular responses [25–27]. *Candida* species are human fungal pathogens, capable of causing both debilitating mucosal infections and life-threatening systemic infections. These species can grow as unicellular yeast, multicellular filaments, or complex biofilm communities [28, 29]. Several *Candida* species have been shown to transition between two phenotypic states, ‘white’ and ‘opaque’, that exhibit marked differences in physical appearance, mating competence, immune cell interactions, filamentous growth, and virulence [30–37]. Bistability between the two forms is achieved by interlocking feedback loops between multiple transcription factors [38–45]. This system has been modeled by a network centered on two transcription factors, Wor1 and Efg1, that are mutually antagonistic to one another [46]. In addition, the white-opaque switch is regulated by both post-translational histone modifications [47–51] and Mediator complex [52], indicating parallels with cell fate decisions in higher organisms.

In this work, we address the mechanism of phenotypic switching in *Candida tropicalis*, and establish that cells exist in three distinct, heritable cell states. In addition to white and opaque, cells propagate in a third “hybrid” form that is intermediate to the two conventional phenotypic states. We dissect the transcriptional regulation of this tristable switch, including the roles of master regulators Wor1 and Efg1 in cell differentiation. We propose that a symmetric self-activating toggle switch (SATS) centered on Wor1 and Efg1 defines the three metastable cell states. In support of this model, we demonstrate that that low, intermediate, and high *WOR1* expression levels drive the formation of white, hybrid, and opaque states, respectively. Conversely, increasing *EFG1* expression drives switching in the opposite direction, from opaque to hybrid to white. Moreover, manipulating ectopic expression of these two genes is sufficient for inducing all six possible cell state transitions in the tristable system.

We also address the heritability of induced cell states in *C*. *tropicalis*. Surprisingly, while ectopic expression of a transcription factor can induce cell state transitions, these transitions are not stably maintained upon turning off ectopic expression, with cells returning to the parental state *en masse*. We therefore examined the potential for chromatin-based cues to impact cell fates. Strikingly, synergistic interactions between gene expression and posttranslational histone modifications were necessary for stable propagation of induced cell states. Together, our results therefore establish a symmetric SATS model for tristable switching in *C*. *tropicalis*, and reveal that coupling between a transcriptional circuit and changes in chromatin structure facilitate heritable cell differentiation. These results allow explicit testing of models of multistable circuits, and underline striking parallels between cell fate determination in unicellular yeast cells and that in metazoan cells.

## Results

### *C*. *tropicalis* exhibits a tristable phenotypic switch

**Fig 1:**
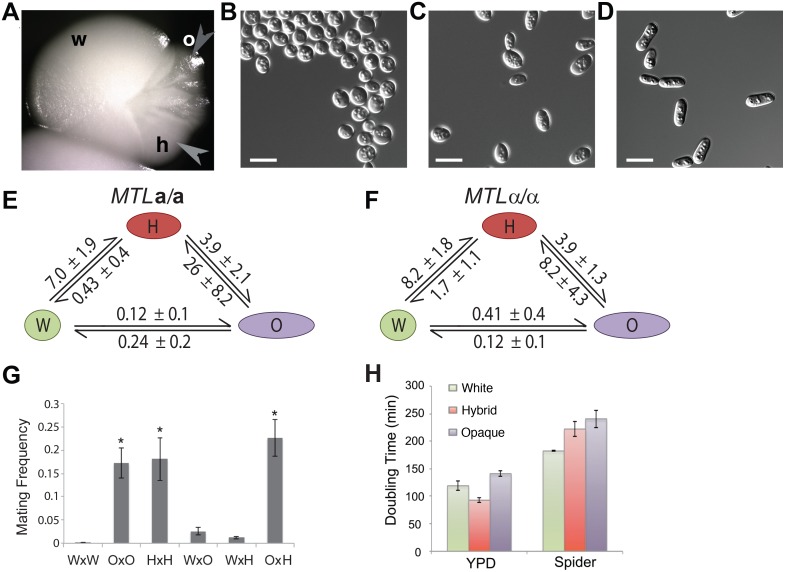
A tristable white-hybrid-opaque switch operates in *C*. *tropicalis*. (**A**) A white colony of *MTL***a**/**a** strain CAY1504 was grown on a Spider plate at 22°C for 10 days. The white (w) colony sectored to hybrid (h, light gray arrowhead) and to opaque (o, dark gray arrowhead). Representative cellular morphologies of (**B**) white cells, (**C**) hybrid cells, and (**D**) opaque cells derived from strain CAY1505. Scale bars = 10 μm. (**E**, **F**) Switching frequencies between the three phenotypic states. (**E**) *MTL***a**/**a** and (**F**) *MTL*α/α strains were grown on Spider medium at 22°C for 10 days and switching frequencies determined. (**G**) Mating frequencies of cells from white (W), opaque (O), and hybrid (H) phenotypes. Mating experiments were performed for 1 day at 22°C on Spider medium. Error bars show standard error, with each data point representing at least 9 replicate experiments. Marked data points (*) are significantly higher than unmarked data points (p<0.001, two-tailed T-test). (**H**) Growth rates for the three phenotypic states. The average doubling time during logarithmic phase growth was determined for white, hybrid, and opaque cells in YPD and Spider media and plotted with standard deviations.

Culturing *MTL* (mating-type like) homozygotes of *C*. *tropicalis* strain ATCC3419 ST-120 revealed three colony types with distinct cellular morphologies (Fig 1A). Switching between the three states was particularly evident in a subset of colonies exhibiting all three phenotypes (Fig 1A). Cells from colonies defined as being in the “hybrid” state exhibited a characteristic ovoid morphology, distinct from that of rounder white cells or more elongated opaque cells (Figs 1B–1D and S1). Testing clinical *C*. *tropicalis* strains identified white, hybrid, and opaque states in four of six strains (including both *MTL* homozygous and *MTL* heterozygous isolates), indicating that tristability is a common attribute of *C*. *tropicalis*.

Transition rates between the three cell states were assessed starting from pure populations of white, hybrid, and opaque cells. White-hybrid and opaque-hybrid switching was significantly more frequent than white-opaque switching, independent of *MTL* configuration (Wilcoxon test (W (30)), p = 0.02), Fig 1E and 1F). For example, in an *MTL***a**/**a** background, white-to-hybrid switching was 7% and hybrid-to-opaque switching was 3.9%, whereas white-to-opaque switching was only 0.12% (Fig 1E). To assess the stability of the hybrid state, single cells were micro-dissected from a hybrid colony and cultured to form new colonies. All of the cells (N = 36) isolated from hybrid colonies re-formed new hybrid colonies (S1 Table). These results establish that cells from hybrid colonies are not white or opaque but represent a truly distinct and heritable third state.

Sexual competency is a key phenotype distinguishing white and opaque cell states in *C*. *tropicalis* [31]. We therefore compared the mating efficiency of *C*. *tropicalis* cells in the white, hybrid, and opaque states. Mating between **a** and α cells in the hybrid state (18.1%) was as efficient as that between opaque cells (17.2%), and more than 100-fold higher than that between white cells (0.13%, p<0.001) (Fig 1G). Hybrid and opaque cells also mated efficiently with one another, but did not mate efficiently with white cells (Fig 1G).

Growth rates of cells in the three states were also compared. In YPD medium at 25°C, hybrid cells grew significantly faster than white cells and both grew faster than opaque cells (Fig 1H; ANOVA; F_(2,6)_ = 47.7, p<0.0002). In contrast, in nutrient-poor Spider medium, white cells grew faster than both opaque and hybrid cells (ANOVA; F_(2,6)_ = 18.1, p<0.003), which grew at similar rates. Taken together, these results indicate that different cell states have distinct phenotypes that impact both cell fitness and sexual fecundity.

### Global gene expression in white, hybrid, and opaque cells

**Fig 2:**
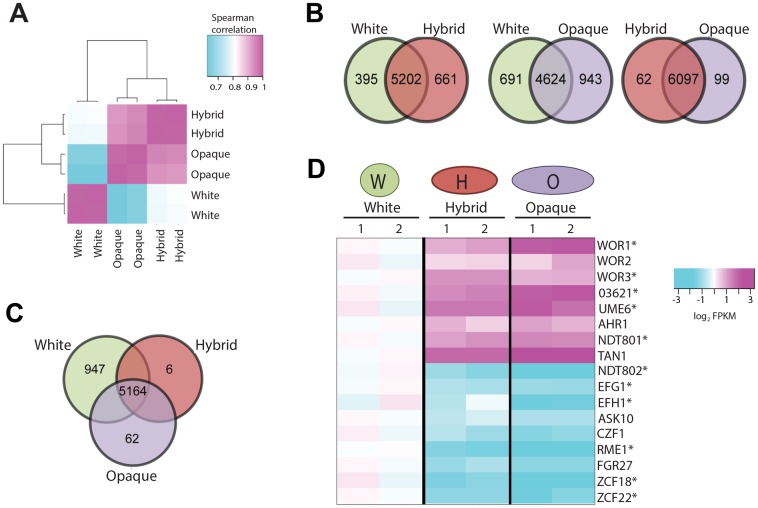
Gene expression profiling of *C*. *tropicalis* cells in the white, hybrid, and opaque states. (**A**) Global comparison of RNA-Seq expression profiles for white, hybrid, and opaque cells. Figure shows the spearman correlation coefficients between -the transcript counts for each sample as a heat map. The two biological replicates for each cell state are highly correlated (0.998–0.999). White and opaque profiles show the least correlation (avg. of 0.202), with a higher correlation between opaque-hybrid (avg. of 0.797) than hybrid-white (avg. of 0.712) profiles. (**B**) Differentially expressed genes (2-fold cutoff and q-value<0.001) between white-opaque, white-hybrid, and hybrid-opaque cell types. (**C**) Differentially expressed genes (2-fold cutoff and q-value<0.001) among the three phenotypic states. (**D**) RNA-seq expression levels of established or putative transcriptional regulators involved in the switch in *C*. *tropicalis* white (CAY3051), hybrid (CAY3393), and opaque (CAY3053) states. Asterisks denote genes expressed at a significantly different level between cell states, and the two columns indicate data from independent experiments.

RNA sequencing (RNA-seq) of *C*. *tropicalis* white, hybrid, and opaque cells revealed unique gene expression profiles for the three states. In general, the expression profile of cells in the hybrid state more closely resembled the profile of opaque cells than white cells (Fig 2A). In fact, only 161 genes were differentially expressed between the opaque and hybrid states (≥2-fold difference, q-value < 0.001) (Fig 2B and S2 Table). In contrast, white cells differentially expressed 1,634 and 1,056 genes when compared to opaque and hybrid cells, respectively (Fig 2B and S2 Table). Each of the three cell states expressed unique gene sets; 947 genes were unique to white cells, 62 genes were unique to opaque cells, and 6 genes were unique to the hybrid state (Figs 2C, S2A and S3 Table).

Gene expression changes linked with key biological processes were identified between the three cell states. Gene Ontology (GO)-term processes significantly associated with white cells included upregulation of redox genes (q < 6.4x10^-14^) and downregulation of ribosome biogenesis and rRNA processing genes (q = 9.0x10^-164^ and 2.2x10^-136^, respectively; S4 Table). Opaque cells upregulated drug transport genes (q = 0.03) and downregulated genes involved in metabolic pathways such as biotin biosynthesis (q = 1.0x10^-5^) and synthesis of carboxylic acid-containing compounds (e.g., monocarboxylic acid metabolism; q = 1.0x10^-4^). Hybrid cells upregulated NADPH genes (q = 2.6x10^-4^) and downregulated oxidoreductase genes (q = 0.005) relative to opaque cells. In fact, genes involved in redox reactions were differentially expressed between all three states (q = 0.01).

*C*. *tropicalis* RNA-seq data was assessed for transcription factors previously found to be differentially regulated between *Candida* white and opaque states [31, 42, 53–55]. Several transcription factors showed increasing or decreasing expression when comparing cells from white, hybrid and opaque states (Fig 2D). For example, *WOR1* and *WOR3* showed a stepwise increase in expression from white to hybrid to opaque cells. For *WOR1*, 12, 23, and 56 fragments per kilobase of transcript per million reads (FPKM) were obtained from white, hybrid, and opaque cells, respectively (Fig 2D, S5 and S6 Tables). Conversely, *EFG1*, the *EFG1* homolog *EFH1*, and *NDT802* were highest expressed in white cells. Six additional *C*. *tropicalis* transcription factors, *ASK10*, *CZF1*, *FGR27*, *RME1*, *ZCF18*, and *ZCF22*, showed elevated expression in white cells, whereas *UME6*, *TAN1*, and *CTRG_03621* showed elevated expression in opaque cells (Fig 2D). Thus, the three cell states express unique sets of genes with a number of differentially expressed transcription factors. Furthermore, comparison of transcriptional profiles between *C*. *tropicalis* and *C*. *albicans* [56] did not reveal a global correlation between white- and opaque-specific expression patterns (S2B Fig), reflecting differences in the transcriptional regulation of gene targets between the two species.

### Genetic analysis of tristable switching

To identify transcription factors that regulate cell fate decisions in *C*. *tropicalis*, deletion mutants were constructed for factors differentially expressed between *C*. *tropicalis* cell states (Fig 2D) or for orthologous genes of the *C*. *albicans* white-opaque circuit. Homozygous deletion mutants were obtained for all targeted genes with the exception of *ZCF22* and *CTRG_03621*, which could represent essential genes. Although previous work has established that deletion of *C*. *tropicalis WOR1* or *EFG1* promotes formation of the white or opaque state, respectively [57, 58], deletion of other tested genes did not significantly affect switching frequencies (e.g., S3 Fig).

**Fig 3:**
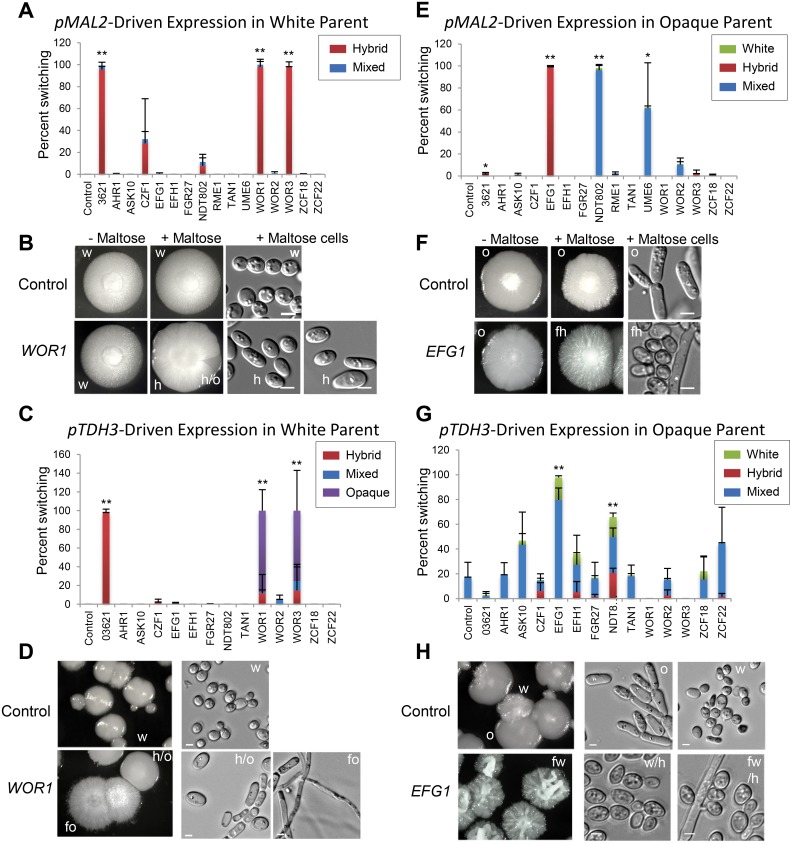
Analysis of *C*. *tropicalis* cells expressing transcription factors under the control of the regulatable *MAL2* or constitutive *TDH3* promoters. Frequency of switching in white parental cells (**A**) or opaque parental cells (**E**) upon *pMAL2*-mediated transcription factor induction (growth on Spider+Maltose medium). * indicates p <0.05, ** indicates p < 0.01 (Student’s t-test). Phenotypes of colonies from white parents (**B**) or opaque parents (**F**) cultured on non-inducing (-Maltose) or inducing (+Maltose) medium at 30°C for 7 days. Cells taken from colonies grown on inducing medium are also shown. Frequency of phenotypic switching in white parental cells (**C**) or opaque parental cells (**G**) upon transformation with transcription factors under *pTDH3* control. ** indicates p < 0.01 (Student’s t-test). Colony morphology (left) and cell morphology (right) from white parental cells (**D**) or opaque parental cells (**H**) transformed with the indicated transcription factor under *pTDH3* control and grown on Spider medium at 30°C for 7 days. Phenotypes are indicated by “o” (opaque), “h” (hybrid), “w” (white), “fo” (filamentous opaque), “h/o” (hybrid/opaque), “fw” (filamentous white) and “fh” (filamentous hybrid). “Mix” refers to a mixture of phenotypes. Scale bars = 5 μm.

Genetic redundancy within transcription factor networks can obfuscate the effects of single gene deletion mutants. We therefore tested whether ectopic expression of candidate regulators altered cell state transitions using the maltose-inducible *MAL2* promoter. *pMAL2*-induced expression of *WOR1*, *WOR3*, or *CTRG_03621* resulted in high rates of white cell switching to the hybrid state (Figs 3A, 3B and S4A). Conversely, *pMAL2*-induced expression of *EFG1*, *NDT802* or *UME6* genes in opaque cells resulted in the majority of these cells switching to the hybrid state or to a mixture of phenotypic states (Figs 3E, 3F and S4B). Interestingly, *pMAL2*-driven overexpression of several white-opaque regulators also increased filamentation in a number of colonies (e.g., *EFG1* in Fig 3F).

Phenotypic switching induced by gene overexpression was also examined using the strong and constitutive *TDH3* promoter [58]. *pTDH3*-mediated overexpression of *WOR1*, *WOR3* or *CTRG_03621* again induced efficient phenotypic switching in white cells (Figs 3C, 3D and S5A). However, whereas *pTDH3-WOR1* or *pTDH3-WOR3* cells exhibited white-to-opaque switching (87.0% and 75.2%, respectively), *pTDH3*-mediated overexpression of *CTRG_03621* almost exclusively induced formation of the hybrid state. Differences in phenotypes observed between *pTDH3* and *pMAL2* promoters presumably reflect differences between weaker *pMAL2-*mediated expression (~7-fold gene induction) and stronger *pTDH3-*mediated expression (~20-fold gene induction) (contrast Fig 3A and 3C).

The effect of *pTDH3*-driven gene expression was also evaluated in opaque cells, although opaque cells were relatively unstable during transformation and often reverted to white or hybrid states. Forced expression of *EFG1* or *NDT802* increased switching from opaque to white/hybrid states (Figs 3G, 3H and S5B). All other tested genes either had no effect or, as in the case of *WOR1*, *WOR3*, and *CTRG_03621*, helped stabilize the opaque state. Ectopic *pTDH3*-driven expression of several transcription factors including *EFG1*, *WOR1* and *WOR3* also increased filamentation in a subset of conditions (S5 Fig).

### Dissection of the transcriptional circuit controlling tristability

**Fig 4:**
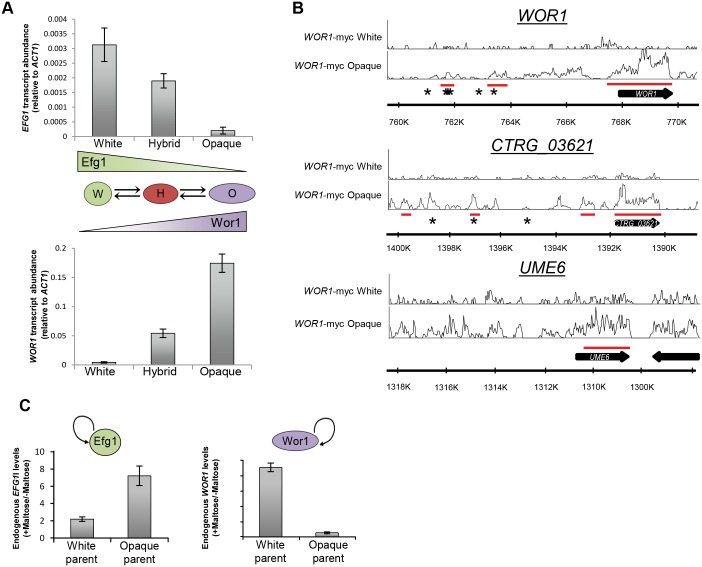
Analysis of the role of Wor1 and Efg1 in *C*. *tropicalis* tristability. (**A**) Total *WOR1* and *EFG1* expression was assayed by qRT-PCR in *C*. *tropicalis* white, hybrid, and opaque cells. Error bars are standard deviations from three replicate experiments. The tristable states are depicted by graded expression of *WOR1* and *EFG1*. In white cells Efg1 expression is high and Wor1 expression is low, in hybrid cells Efg1 and Wor1 are expressed at intermediate levels, and in opaque cells Wor1 expression is high and Efg1 expression is low. (**B**) Determination of Wor1 binding to genomic regions in white and opaque cells using ChIP-Seq. DNA binding by Wor1 protein was observed at discrete sites within the *WOR1* and *CTRG_03621* promoters, as well as within *WOR1*, *CTRG_03621*, and *UME6* ORFs in opaque cells. The y-axis represents density of coverage. Underlined regions in red indicate regions of significant binding compared to the untagged control (see Materials and Methods). Asterisks denote positions corresponding to the *C*. *albicans* Wor1 DNA binding motif. (**C**) Transcript abundance of the endogenous *WOR1* or *EFG1* transcript when ectopic *pMAL2-WOR1* or *pMAL2-EFG1* was induced, respectively. Relative expression changes were determined between inducing conditions (+Maltose) and non-inducing conditions (-Maltose) and demonstrate auto-activation by each factor.

The results described above indicate that Wor1 and Efg1 play prominent but contrasting roles in regulating *C*. *tropicalis* cell states. To further examine their behavior in cell differentiation, *WOR1* and *EFG1* expression levels were compared between white, hybrid, and opaque cells by quantitative RT-PCR (qRT-PCR). *WOR1* transcript levels cells increased by 7- and 34-fold in hybrid and opaque cells, respectively, relative to white cells (Fig 4A). In contrast, *EFG1* transcript levels decreased by 1.7- and 15-fold in hybrid and opaque cells relative to white cells (Fig 4A). Thus, *WOR1* and *EFG1* expression levels reveal an inverted relationship; *WOR1* exhibits a stepwise increase in expression from white to hybrid to opaque cells, whereas *EFG1* shows a stepwise decrease in expression between these same cell types.

The opaque state in *C*. *albicans* is stabilized by positive auto-regulation of Wor1 acting on its own promoter [38–40]. To test if a similar feedback loop operates in *C*. *tropicalis*, chromatin immunoprecipitation and DNA sequencing (ChIP-seq) was performed using an epitope-tagged *WOR1* allele. Wor1 binding was significantly enriched at 54 genomic regions across 48 loci in opaque cells, whereas it did not show any significant binding to DNA in white cells (Fig 4B and S7 Table). Binding was enriched among genes involved in mating projection formation (q = 0.04) and to genes encoding transcriptional regulators of the phenotypic switch (Figs 4B and S6; p<0.001, χ^2^). Wor1 localized to discrete sites both upstream of and within the *WOR1* ORF, supportive of auto-regulation of the endogenous gene. Significant levels of Wor1 enrichment were also observed in the promoter and/or ORFs of *UME6* and *CTRG_03621*, two transcription factors that also regulate cell switching (Fig 4B).

In general, the bulk of *C*. *tropicalis* Wor1 binding was found contiguously within target ORFs rather than in promoter regions (Figs 4B and S6). Analysis of sequences significantly enriched in Wor1 binding sites identified a motif that showed similarity to the binding motif of Azf1 (p = 4.3x10^-5^), a zinc finger transcription factor involved in nutrient sensing in *S*. *cerevisiae* [59]. *C*. *albicans* Wor1 recognizes a 14-bp motif [60], but this motif was not significantly enriched among *C*. *tropicalis* Wor1-bound regions, although it was present in 13 out of 54 of these regions. Given conservation of the DNA binding specificity of Wor1 orthologs across diverse fungal species [61], it is likely that *C*. *tropicalis* Wor1 binds a number of target genes indirectly via interactions with other transcriptional co-factors, potentially including the ortholog of Azf1 (*CTRG_00920*).

Mutual activation/mutual inhibition (MAMI) circuits are common regulatory features in defining cellular states. While mutual antagonism between transcription factors can give rise to bistability, autoregulation of at least one of these transcription factors allows a system to adopt additional steady states including tristability [16, 20, 21]. Tristability has been modeled by a symmetric self-activating toggle switch (SATS) in which both transcription factors are self-activating but mutually antagonistic [10, 16, 20]. A SATS model of tristability could apply to *C*. *tropicalis*, in which the key transcription factors Wor1 and Efg1 would be both positively auto-regulatory and mutually antagonistic.

To determine whether Wor1 and Efg1 exhibit auto-activation, ectopic expression of *WOR1* and *EFG1* was induced in white and opaque cells using the *pMAL2* promoter, and expression levels of the endogenous genes determined by qRT-PCR. Induction of *WOR1* in white cells increased expression of the endogenous *WOR1* gene and, conversely, induction of *EFG1* in opaque cells induced endogenous *EFG1* expression (Fig 4C). This establishes that both Wor1 and Efg1 positively auto-regulate their own gene expression, consistent with the symmetric SATS model. In the case of Wor1, auto-regulation is likely direct given that Wor1 binds to its promoter and ORF sequence. In summary, expression levels of *WOR1* and *EFG1* show an inverse correlation between cell states consistent with mutually antagonistic activities (Fig 4A), and are both auto-activating as in a symmetric SATS (Fig 4C).

### Testing a SATS model for tristability

**Fig 5:**
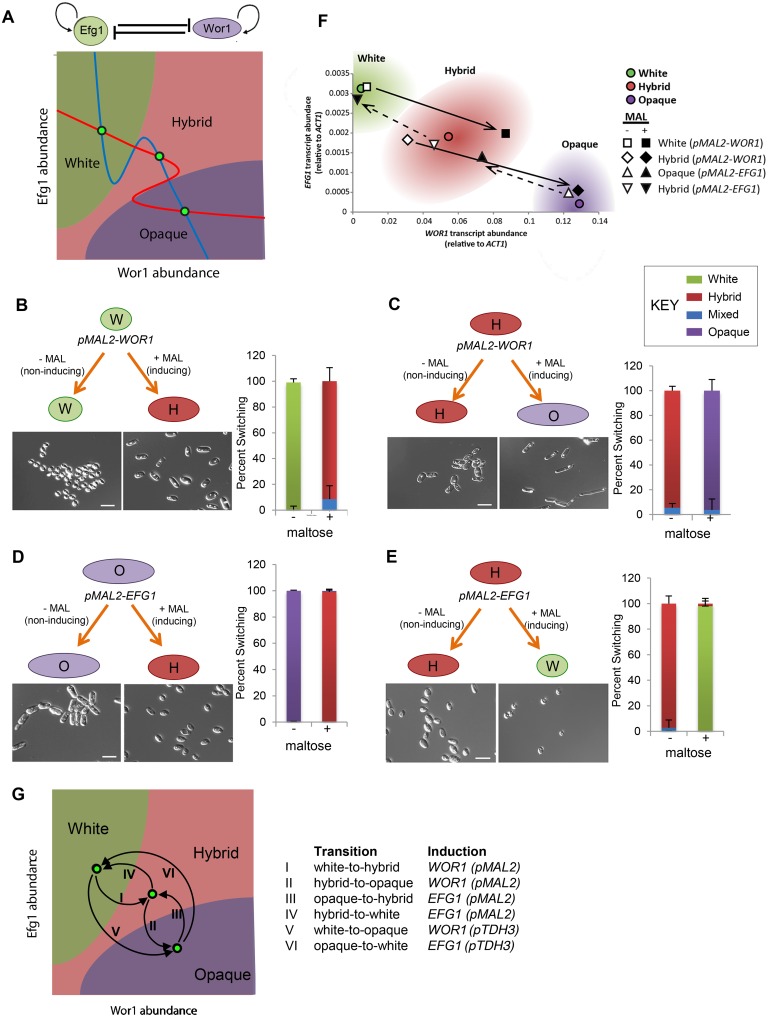
Testing a symmetric SATS model of tristability. (**A**) Model of a self-activating toggle switch (SATS) that is proposed to operate between Wor1 and Efg1. The nullclines (lines in the x-y planes) are plotted for Wor1 (red) and Efg1 (blue) based on the SATS model. Intersection of the nullclines (green circles) indicate stable states corresponding to the white, hybrid, and opaque phenotypic states. Regions of the Wor1/Efg1 spacescape are color coded according to the predicted resulting phenotypic state. (**B**-**E**) To test this model, *pMAL2-WOR1* (**B**, **C**) or *pMAL-EFG1* (**D**, **E**) were ectopically expressed in different cell states. Cells containing these constructs were plated onto media containing maltose (+MAL, inducing conditions) or lacking maltose (-MAL, non-inducing conditions) and grown at 30°C for 7 d. Phenotypes were defined by microscopic analysis of cell morphologies (see representative cell pictures) and switching frequencies calculated (see graphs). Scale bar, 10 μm. Error bars, standard deviation. “Mixed” refers to colonies with a mixture of cellular phenotypes. (**F**) *WOR1* and *EFG1* transcript levels define *C*. *tropicalis* white, hybrid, and opaque states. Transcript levels for control cells of each state are depicted as colored circles. Cells containing the *pMAL2-WOR1* or *pMAL2-EFG1* construct are depicted as either open (-MAL) or filled (+MAL) symbols. Arrows indicate the shift in *WOR1* and *EFG1* levels following induction of either *pMAL2-WOR1* or *pMAL2-EFG1*. *WOR1* was induced in white and hybrid states, whereas *EFG1* was induced in hybrid and opaque states. (**G**) Schematic summarizes how six different transitions between white, hybrid, and opaque states can be induced by ectopic expression of *WOR1* and *EFG1* genes.

Applying a symmetric SATS model to *C*. *tropicalis*, the white state can be defined by high Efg1/low Wor1 expression, the opaque state by high Wor1/low Efg1 expression, and the hybrid state by intermediate expression of both transcription factors (Fig 5A). To test this model, we examined the effect of ectopic *WOR1* and *EFG1* expression on cell identity when induced in different states. The *pMAL2-WOR1* construct was separately introduced into white and hybrid cells, and transformed cells grown on inducing and non-inducing media. Significantly, ectopic expression of *WOR1* in white cells resulted in efficient switching (91%) to the hybrid state, whereas induction of *WOR1* in hybrid cells resulted in efficient switching to the opaque state (96%, Fig 5B and 5C). No switching was observed under non-inducing conditions in either white or hybrid cells. These results are striking as they establish that ectopic expression of the same gene can drive alternative cell fates dependent on the starting state of the cell. Thus, *WOR1* expression in the white state induces white-to-hybrid switching, whereas expression in the hybrid state induces hybrid-to-opaque switching.

We similarly tested ectopic *EFG1* expression in different starting cell states. Induced expression of *pMAL2-EFG1* resulted in efficient opaque-to-hybrid switching (99%, Fig 5D), while induction of the same gene in hybrid cells induced switching to the white state (97%, Fig 5E). These results establish that *WOR1* and *EFG1* drive cell differentiation events in opposite directions, and that induced cell states are critically dependent on the starting state of the cell.

Ectopic expression of *pMAL2-WOR1* also altered the expression levels of both *WOR1* and *EFG1* in line with the new cell state. Induction of *pMAL2-WOR1* expression in white cells resulted in a 11-fold increase in *WOR1* levels (includes both ectopic and endogenous *WOR1*) and a 1.6-fold decrease in *EFG1* levels, consistent with the expression levels of these genes in naturally occurring hybrid cells (Figs 5F and S7). Similarly, ectopic *WOR1* induction in hybrid cells increased *WOR1* expression and decreased *EFG1* expression to levels similar to that in naturally occurring opaque cells (Fig 5F). *pMAL2-*induced *EFG1* expression also produced *WOR1* and *EFG1* expression patterns that corresponded to defined cell states. Thus, ectopic induction of *EFG1* in opaque cells produced *WOR1* and *EFG1* levels corresponding to the intermediate state, whereas ectopic expression in hybrid cells produced *WOR1* and *EFG1* levels that corresponded to cells in the white state (Fig 5F). Transitions between each of the different cell states are therefore possible simply by regulating the expression of *WOR1* and *EFG1* (Fig 5G).

Together, our observations provide several lines of experimental support for a symmetric SATS model of tristability operating in *C*. *tropicalis*: (1) A stepwise increase in *WOR1* expression is observed between the three phenotypic states, (2) A corresponding stepwise decrease in *EFG1* expression levels is observed between states, (3) Wor1 and Efg1 show positive auto-regulation of their own genes but antagonism towards the opposing factor, (4) Ectopic *WOR1* expression can induce white-to-hybrid, hybrid-to-opaque, or white-to-opaque transitions, dependent on the parental state of the cell and the strength of *WOR1* induction, (5) Ectopic *EFG1* expression induces switching in the opposite direction, resulting opaque-to-hybrid-to-white switching, again dependent on the strength of the promoter (*pMAL2* or *pTDH3*) and the parental cell state.

### Regulation of heritable cell fates by synergistic transcriptional and chromatin-based cues

**Fig 6:**
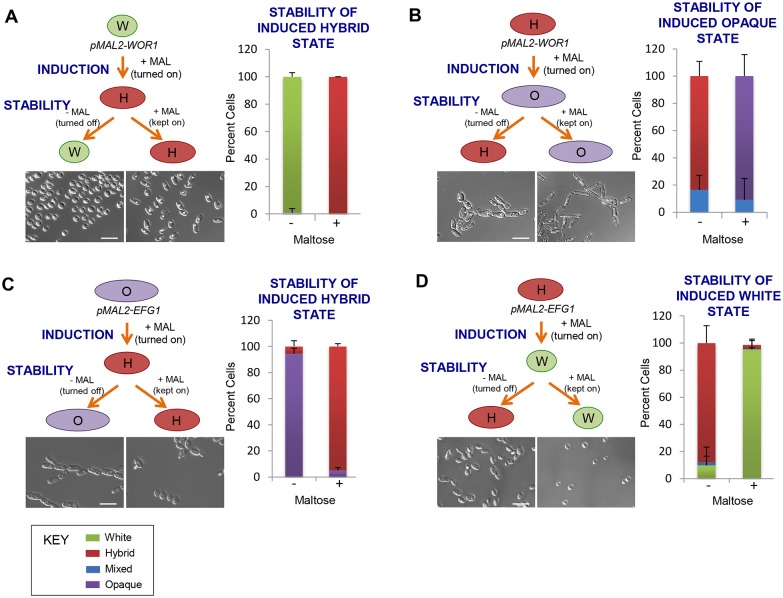
Stability of induced phenotypic states. To determine the stability of phenotypic states induced by ectopic expression of *pMAL2*-*WOR1* (**A**,**B**) or *pMAL2-EFG1* (**C**,**D**), cells were taken from inducing conditions (+MAL) and re-plated to media either with or without maltose. Colony phenotypes were examined after 7 d growth at 30°C. Scale bar, 10 μm. Error bars, standard deviation. “Mixed” refers to colonies with a mixture of cellular phenotypes.

The experiments described above establish that ectopic gene expression can drive efficient differentiation between all three cell states in *C*. *tropicalis*. However, we found that shutting off ectopic expression consistently resulted in the majority of cells returning to the parental state, independent of the transcription factor utilized. For example, ectopic expression of *pMAL2*-*WOR1* resulted in white cells switching to the hybrid state, but turning off ectopic expression resulted in cells returning to the white state *en masse* (99% of cells returned to white, Fig 6A). The same was true for ectopic expression of *pMAL2*-*WOR1* in hybrid cells; these cells efficiently switched to the opaque state, but 91% of these cells returned to the hybrid state upon turning off ectopic expression (Fig 6B). Similar results were observed using the *pMAL2-EFG1* construct, as ectopic *EFG1* expression induced opaque-to-hybrid or hybrid-to-white switching, but shutting off ectopic expression resulted in cells returning to the parental state (Fig 6C and 6D). Ectopic expression of other transcription factors also failed to drive stable changes in cell state, so that states reverted once ectopic expression was turned off (S8 Fig).

Changes in chromatin structure are closely associated with heritable cell states in multiple species, and post-translational histone modifications were previously shown to regulate white-opaque switching in *C*. *albicans* [47–51]. To determine if histone modifications also impact cell states in *C*. *tropicalis*, we examined the effect of nicotinamide (NAM), a sirtuin histone deacetylase (HDAC) inhibitor, on phenotypic switching. Addition of NAM to the medium induced a a dose-dependent change in white and hybrid cell states, with cells switching to hybrid and opaque states, respectively (S9A and S9B Fig). However, as with induced expression of transcriptional regulators, removal of cells from NAM-containing medium resulted in switching back to the original cell state *en masse*. This result establishes that chromatin-mediated signals can induce phenotypic switching in *C*. *tropicalis* similar to forced transcription factor expression, but that neither of these stimuli alone are sufficient for stable maintenance of induced cell states.

**Fig 7:**
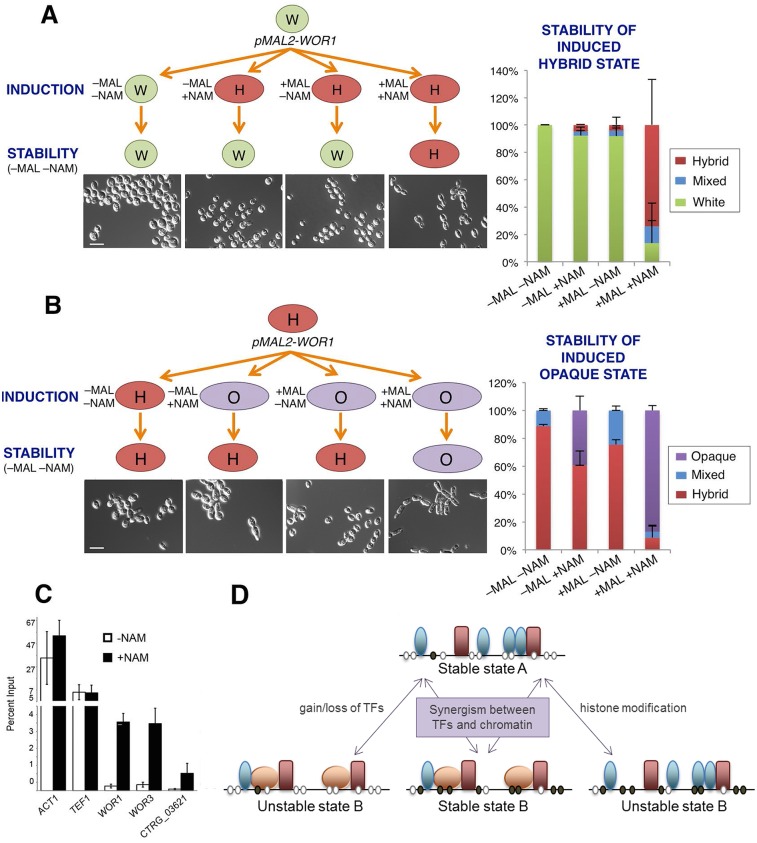
Stable switching requires both ectopic *WOR1* expression and changes in chromatin structure. Cells encoding an ectopic *pMAL2-WOR1* construct in (**A**) white cells or (**B**) hybrid cells were plated onto inducing (+MAL) or non-inducing (-MAL) medium. In addition, some plates were supplemented with 5 mM NAM (a histone deacetylase inhibitor). Colonies were grown at 30°C for 7 days before re-plating to media lacking both maltose and NAM and grown for a further 7 days at 30°C. Colony phenotypes were determined and quantified. (**C**) White cells were grown either in the presence or absence of 5 mM NAM at 30°C for 24 hours. Chromatin immunoprecipitation was performed for H3K56Ac in induced and uninduced cultures. Quantification of H3K56Ac enrichment at cell state regulators and control genes was performed by qPCR and compared to input controls for three biological replicates. (**D**) Model of the dual regulation of epigenetic switching by transcriptional activation and histone modification. Induction of either stimulus alone is capable of inducing state switching, but loss of the stimulus results in cells reverting to the original state. Cells experiencing both transcriptional activation and chromatin remodeling (via HDAC inhibition) are able to stably maintain the new cell state even after the removal of switch-inducing factors.

Next, we investigated whether transcriptional and chromatin-based cues could act cooperatively to drive stable switching. *C*. *tropicalis* cells containing the *pMAL2*-*WOR1* construct were cultured in inducing (+MAL) or non-inducing (-MAL) conditions, as well as in the presence or absence of 5 mM NAM. Cells were subsequently re-cultured without either of these stimuli to determine the stability of induced states. Significantly, only cells that ectopically expressed *WOR1* and were co-exposed to NAM stably retained the inherited cell state after stimuli were removed (Fig 7A and 7B). This was particularly evident for cells induced to switch from the white state to the hybrid state. Whereas ectopic expression of *WOR1* or the inclusion of NAM resulted in only 4–5% of white cells stably transitioning to and maintaining the hybrid form, inclusion of both stimuli resulted in 76% of cells retaining the hybrid state (Fig 7A). Furthermore, the induced hybrid state was stable for multiple generations upon subsequent passaging (S9C Fig).

Treating cells with NAM has previously been shown to increase acetylation of H3K56, a mark associated with increased histone turnover and elevated expression of adjacent genes [48, 62, 63]. To determine if chromatin alterations occurred following exposure to NAM in *C*. *tropicalis*, we assessed H3K56 acetylation levels at several genetic loci in the presence and absence of NAM. White cells treated with NAM showed increased abundance of H3K56Ac at the promoters of transcription factors *WOR1*, *WOR3*, and *CTRG_03621* that promote the opaque state, but did not influence H3K56Ac at control promoters (Fig 7C). Together, these results indicate that transcriptional and chromatin-based signals act synergistically to drive heritable differentiation events in *C*. *tropicalis*.

## Discussion

In this paper, we use a combination of genetic and genomic approaches to address the mechanism of multistate switching in *C*. *tropicalis*. Our data establishes that *C*. *tropicalis* undergoes heritable and reversible switching between three stable cell states–white, hybrid, and opaque. We provide experimental support for a model in which the two master transcription factors, Wor1 and Efg1, define a symmetric self-activating toggle switch to produce tristability. Furthermore, we demonstrate that transcriptional activators work synergistically with chromatin-based cues to drive heritable differentiation events.

The three phenotypic states observed in *C*. *tropicalis* were examined in depth. Each cell type could be stably propagated for multiple generations, with stochastic interconversion observed between all three states. Transcriptional analysis revealed that hybrid cells exhibited a profile closer to that of opaque cells than white cells and, consistent with this observation, hybrid cells mated with a similar efficiency to opaque cells. However, each cell type still displayed a state-specific expression profile, which included differential expression of redox and carbon metabolism genes. Metabolic differences could account for distinct growth rates between the three states and may also have consequences for growth in the host. In the related human pathogen *C*. *albicans*, white and opaque cells exhibit marked differences in metabolism that likely contribute to the preferential colonization of different host niches by each cell type [34, 64, 65].

Bistability in natural systems is often mediated by a toggle switch, in which two mutually antagonistic transcription factors compete to establish one of two alternative cell fates [20, 66]. Indeed, a toggle switch has previously been proposed to regulate bistable white-opaque switching in *C*. *albicans* [46]. These circuits typically demonstrate robust bistability and hysteresis, which reinforces the acquired cell state. Modeling indicates that one or both components of the circuit must exhibit auto-regulation in order for a two-component system to generate more than two stable cell states [10, 16, 17, 20]. In a symmetric self-activating toggle switch (symmetric SATS) both components display self-activation and these systems are predicted to maintain tristability across a range of inputs and interaction strengths [16, 20]. However, an inability to study multistability in experimentally tractable systems has hindered direct testing of these models.

In this work, we establish that a symmetric SATS model centered on Wor1 and Efg1 transcription factors defines tristable switching in *C*. *tropicalis*. Here, the white state is defined by high Efg1/low Wor1 expression, the hybrid state by mid-level Efg1/Wor1 expression, and the opaque state by low Efg1/high Wor1 expression (Fig 4A). In line with this model, expression levels of *WOR1* and *EFG1* were inversely correlated; *WOR1* increased from white to hybrid to opaque states, whereas *EFG1* expression decreased between these same three states. In addition, these two factors are mutually inhibitory, as ectopic induction of *WOR1* and *EFG1* inhibited expression of the other. Furthermore, as required for a symmetric SATS, both Wor1 and Efg1 positively auto-regulated their own gene expression.

Crucially, we were able to manipulate transitions between all three tristable states by forced expression of either *WOR1* or *EFG1*. Thus, ectopic *WOR1* expression induced both white-to-hybrid and hybrid-to-opaque switching, depending on the starting state of the cell, and stronger expression of *WOR1* resulted in white-to-opaque switching, effectively bypassing the hybrid state. Conversely, ectopic *EFG1* expression induced switching in the opposite direction and was capable of forcing opaque-to-hybrid, hybrid-to-white, and opaque-to-white transitions. Both *WOR1* and *EFG1* are therefore capable are driving phenotypic switching between multiple cell states, dependent both on the level of gene expression and the state in which expression is activated. These results indicate that cell states in *C*. *tropicalis* involve the discrete expression of regulatory factors at multiple levels instead of a Boolean model of expression. This is, to our knowledge, the first time that gene expression has been successfully used to drive each of the six possible cell state transitions present in a tristable system (Fig 5G). Our studies establish that *C*. *tropicalis* is a tractable system for modeling and experimental manipulation of multistability.

Recent studies in *C*. *albicans* have also revealed the existence of phenotypic states related to, but distinct from, conventional white and opaque forms [67, 68]. For example, several *C*. *albicans* strains were shown to undergo a tristable switch between white, opaque and “gray” states [68]. Interestingly, regulation of the gray state shows that it is distinct from the hybrid state in *C*. *tropicalis*. Thus, whereas *C*. *albicans* cells were locked in the gray state in the absence of Wor1 and Efg1 [68], both of these transcription factors promote formation of the hybrid state in *C*. *tropicalis*. Orthologs of the same transcription factors therefore regulate phenotypic switching in the two *Candida* species, but define different cell states due to alternative wiring of the circuits. As further evidence for differences in the regulation of cell identity between species, we note that only four components of the circuit regulate white-opaque switching in both *Candida* species (Wor1, Wor3, Efg1, and OfiI) [41, 69]. In contrast, Ndt802 and Ume6 regulate phenotypic switching only in *C*. *tropicalis*, whereas Ahr1, Czf1, and Wor2 appear to regulate switching only in *C*. *albicans* [42, 53].

Our studies also addressed the inheritance of epigenetic states in *C*. *tropicalis*. Ectopic expression of any one of several transcription factors induced a change in cell state, but turning off ectopic expression resulted in cells returning *en masse* to the parental state. Post-translational modifications have been implicated in regulating the frequency of white-opaque switching in *C*. *albicans*, including a role for acetylation of histone H3K56 in formation of the opaque cell type [47–51]. We show that NAM, a histone deacetylase (HDAC) inhibitor, similarly impacts cellular transitions in *C*. *tropicalis*. Addition of NAM increased H3K56 acetylation at the promoters of transcription factors critical for phenotypic switching, and also caused white cells to transition towards hybrid or opaque states. Notably, however, cell states induced by NAM were again unstable, with cells reverting to the parental state if NAM was removed. In contrast, cells maintained the new phenotypic state when inducing cues were used in combination. Thus, ectopic expression of *WOR1* together with the HDAC inhibitor successfully induced stable white-to-hybrid and hybrid-to-opaque switching (Fig 7).

Studies in metazoans have similarly demonstrated that transcriptional and chromatin-based signals can act in concert to drive heritable epigenetic transitions. For example, the reprogramming of somatic cells into induced pluripotent stem (iPS) cells was initially achieved via the ectopic expression of four transcription factors [70]. Subsequent studies included chromatin modifiers such as DNA demethyltransferase or HDAC inhibitors to increase efficiency and reduce the number of transcription factors required for somatic reprogramming [71–74]. Our results therefore reveal striking parallels between the regulation of dedifferentiation events during somatic cell reprogramming and those accompanying phenotypic switching in yeast. In both systems, heritable cell fate decisions can be induced by the synergistic action of transcription factors acting together with chemically induced changes in chromatin modifications.

In summary, we present and experimentally test a model for multistate switching in unicellular yeast. Our studies indicate that a symmetric SATS regulates tristability in *C*. *tropicalis* and reveal a tractable system for dissection of multipotency. Furthermore, these experiments are the first to reveal that chromatin- and transcriptional-based cues act synergistically to drive stable differentiation events in a microbial system. Given parallels with cell differentiation in higher organisms, it is evident that cellular fates are controlled by related mechanisms in diverse eukaryotic species. It also reveals that phenotypic switching in unicellular yeasts can provide fundamental insights into cell fate determination, with implications for understanding differentiation and reprogramming in higher eukaryotes.

## Materials and Methods

### Media

Media was prepared as previously described [75, 76]. Yeast extract peptone dextrose (YPD) plates or Spider plates containing 200 μg/ml nourseothricin (NAT) were used for selection of strains that were resistant to nourseothricin (SAT^R^ strains) [77]. For induction of genes under the *MAL2* promoter, Spider medium was made without mannitol and supplemented with 2% maltose.

### Strain/plasmid construction

All strains, plasmids and oligonucleotides used in this study are listed in S8, S9 and S10 Tables, respectively. Transformations were performed as previously described for *C*. *tropicalis* [31]. Nutritional gene deletions were constructed using the *SAT1* flipper strategy [77]. Plasmids to delete *HIS1* and *ARG4* were made as described [31, 58]. Deletion of transcription factors was performed using a previously described method [78]. 5’ and 3’ regions flanking the ORF were PCR amplified. *HIS1* and *ARG4* auxotrophic markers were PCR amplified from plasmids pSN52 and pSN64 [78], respectively. Fusion PCR was then performed to fuse 5’ and 3’ flanks to nutritional markers. This PCR product was transformed into auxotrophic strains lacking *HIS1* and *ARG4*. PCR was used to confirm correct genomic integration, the *SAT1* marker removed by recombination [77], and the process repeated to delete the second copy of the ORF. PCR was performed to confirm deletion of the target gene.

To overexpress transcription factors using *pTDH3*, target genes and the *TDH3* promoter were amplified by PCR and the two fragments joined by fusion PCR. The fusion products were cloned into pSFS2a [77] using restriction enzymes ApaI and XhoI. The plasmids were linearized in the *TDH3* promoter by cutting with SmaI or PacI and transformed into *C*. *tropicalis*. Correct genomic integration at the *TDH3* promoter was confirmed by PCR. To express transcription factors under the *MAL2* promoter, plasmid pRB324 was constructed by cloning the *C*. *tropicalis MAL2* promoter into pSFS2a using KpnI and ApaI. PCR was used to amplify transcription factors that were then cloned into this plasmid using ApaI/XhoI or ApaI/BamHI, as noted in S9 Table. Plasmids were linearized in the *MAL2* promoter using a partial digest with PvuI. Correct integration at the *C*. *tropicalis MAL2* promoter was confirmed by PCR.

### Mating assays

Quantitative mating analyses were performed as previously described [31]. Briefly, cells were taken from white, hybrid and opaque phenotypic states after growth on Spider medium and resuspended in water. Approximately 1 x 10^7^ cells of an *MTL***a** and an *MTL*α strain were mixed and spotted onto 0.8 μm nitrocellulose filters on the surface of Spider plates. Plates were incubated at 22°C for 1 day and cells recovered and plated at different dilutions onto His- Arg- medium (to select for mating products) and onto His- and Arg- media (to determine parental populations). The overall mating efficiency was calculated as: mating efficiency = conjugants/(limiting parent + conjugants) = the greater of (Arg- His-)/Arg- or (Arg- His-)/His-.

### Microscopy

Digital images of colonies were collected using a Zeiss Stemi 2000-C microscope equipped with an Infinity 2 digital camera and Infinity Analyzer software (Lumenera Corporation, Ottawa, Canada). Differential interference contrast (DIC) images of cells were captured using a Zeiss Inverted Microscope (Axio Observer) fitted with an AxioCam HR. Images were processed with AxioVision Rel. 4.8 (Zeiss, Germany). To compare cellular phenotypes, eight or more images containing >250 cells were analyzed using CellProfiler v2.1.1 (Broad Institute of MIT and Harvard, Cambridge, MA) for each phenotype. Images were processed in the following manner: Edges were enhanced using the Sobel method, then a threshold was applied and cells identified using the mixture of Gaussian method. The eccentricity, form factor, and ratio of maximum and minimum Feret diameters of each cell were then calculated. Average and standard error were calculated using Microsoft Excel.

### Growth assays

White (CAY3051), hybrid (CAY3393), and opaque (CAY3053) cells derived from strain CAY1505 (*MTL*α/α) were grown at 25°C in liquid Spider medium overnight. The cultures were then diluted 1:200 into either fresh YPD or Spider medium. Optical density was measured every 15 minutes for 48 hours with a plate reader (Tecan) and the polynomial measurement of the curve was used to derive doubling times.

### RNA extraction and RNA-Seq

White (CAY3051), hybrid (CAY3393), and opaque (CAY3053) cells derived from strain CAY1505 (*MTL*α/α) were grown at 25°C in liquid Spider medium to 1.6–1.8 OD_600_. RNA was isolated using the Ribopure-Yeast Kit (Ambion). RNA was treated with Turbo DNaseI (Ambion). RNA quality was measured on an Agilent 2100 Bioanalyzer at the Brown University Genomics Core Facility, and RNA with RIN scores ≥7 used for RNA-Seq.

PolyA RNA was isolated and used to construct strand-specific libraries using the dUTP second strand marking method [79, 80] as previously described [81]. Samples were pooled and sequenced on the Illumina HiSeq to generate 101 base reads. To measure gene expression, reads were aligned to the *C*. *tropicalis* MYA-3404 genome. RNA-Seq reads were then mapped to the transcripts with Tophat2 (version 2.0.9) [82], and placement of multiply mapped reads was estimated using RSEM (version 1.1.18) [83]. Differentially expressed genes were identified using CuffDiff (Cufflinks version 2.1.1) [84]. RNA-Seq data is available online and links are provided in S11 Table. Gene Ontology (GO) analysis was performed using the GO term finder incorporated into the Candida Genome Database (http://www.candidagenome.org/cgi-bin/GO/goTermFinder) [85] with corrections for multiple hypothesis testing.

### Isolation of single cells for phenotypic stability

Cells from a white, hybrid, or opaque colony were spread onto a plate of Spider medium. Single cells were picked using a micromanipulator and individually moved so that the resulting colonies could be analyzed for phenotype. For hybrid cells, a distribution of cell shapes (from round to elongated) were picked from hybrid colonies. Cells were allowed to grow at 30°C for 7 days and phenotypes analyzed by colony and cellular morphology.

### Phenotypic switching assays on *C*. *tropicalis* mutant strains

Strains were grown overnight at 30°C in Spider medium. Cells were diluted in water and plated onto Spider medium at a concentration of ~100 colonies per plate. Plates were incubated for 2 days at 30°C and for 8 additional days at 22°C or 30°C and colonies examined for sectors.

### Phenotypic switching assays for strains expressing genes under the control of the *MAL2* promoter

Strains in the white or opaque state were inoculated from Spider plates into liquid Spider and grown overnight at 30°C. Cells were diluted in water and plated to Spider medium or Spider+Maltose medium to produce ~100 colonies/plate. Plates were incubated at 30°C for 7 days and phenotypes analyzed.

### Phenotypic switching assays for strains expressing genes under the control of the *TDH3* promoter

White (CAY4599) and opaque (CAY4600) cells were grown at 30°C for 5 hours in YPD liquid. Cells were checked for phenotypic purity and inoculated into YPD liquid and grown at 22°C overnight. These cells were transformed with plasmids containing target transcription factors under the *TDH3* promoter, as well as a control plasmid containing *TDH3*, as previously described [31]. Cells were plated to Spider medium containing 200 μg/mL nourseothricin and incubated at 30°C for 7 days. Phenotypes were analyzed and replica patched to Spider plates for PCR testing. Correct integration of constructs at the *TDH3* locus was confirmed by PCR.

### Stability of ectopically induced states

Experiments were performed by one of two ways. In Fig 2, strains containing *pMAL2*-driven target genes were grown on Spider+Maltose plates to induce expression of transcription factors. Colonies were inoculated into Spider+Maltose liquid medium and grown at 30°C overnight. Cells were diluted in water and plated to Spider+Maltose or regular Spider media at a concentration of ~100 colonies/plate. Plates were incubated at 30°C for 7 days and phenotypes analyzed. In Figs 4 and 5, cells were taken from Spider plates, diluted in H_2_O, and plated for single colonies on Spider, Spider+Maltose, Spider+5 mM nicotinamide, or Spider+Maltose+5 mM nicotinamide plates. After 7 days at 30°C, colony phenotypes were determined by analysis of cell morphologies. For stability of the induced states, cells were taken from these plates, diluted in H_2_O, and plated for single colonies on inducing or non-inducing media. After 7 days at 30°C, colony phenotypes were again analyzed by cell morphologies.

### RT-PCR analysis

RNA was isolated from ~2 × 10^8^ cells grown on Spider medium for 4 h either in the presence or absence of maltose. Cells were removed from the medium and RNA isolated using the Ribopure-Yeast Kit (Ambion). RNA was treated with Turbo DNase (Ambion), and 500 ng of RNA used for cDNA generation using the GoScript enzyme (Promega). qRT-PCR was then performed by using primers in S10 Table. cDNA levels were normalized to *ACT1* expression levels.

### ChIP-Seq methods and analysis

White and opaque cells from *WOR1*-myc tagged strains (CAY5955 and CAY5956, respectively) as well as untagged controls (CAY4599 and CAY4600) were grown at 22°C overnight. Purity of each cell state was verified by microscopy prior to sample preparation. Chromatin immunoprecipitation (ChIP) was performed as described previously [86] using an anti-myc antibody (clone 4AG, Millipore, Billerica, MA). Immunoprecipitated DNA was prepared for ChIP-Seq libraries using in-house designed adapters analogous to the Illumina TruSeq system, and based on the Illumina sample preparation guidelines for TruSeq ChIP Sequencing of DNA (Illumina, San Diego, CA). The resulting libraries were sequenced on a HiSeq 2500 platform at Washington University’s GTAC sequencing facility. Demultiplexed reads were assayed for quality using FastQC [87]. Reads were aligned to the *C*. *tropicalis* MYA-330 reference genome using Bowtie2 [88] with the default settings. Read alignment was visualized using the Integrated Genome Viewer (IGV, The Broad Institute, Cambridge, MA) and peak detection was performed using Model-based Analysis of ChIP-Seq (MACS2) [89] using a four-fold change and 10^−3^ critical value cutoff compared to the untagged control strain for each cell state. Reproducibility was compared using an irreproducibility discovery rate function. Motif enrichment was performed by extracting the corresponding peak sequences using Bedtools and using Multiple Em for Motif Enrichment (MEME; version 4.9.1)[90]. Our analysis identified a motif, TKYWKHTKKTKHTTTTTKTTKYTTTKWTT, significantly enriched (p = 7.0x10^-45^) in Wor1 binding sites. This motif occurred multiple times within Wor1-bound regions, spaced by intervals as short as 6 nucleotides. Motifs were commonly clustered at the center of the Wor1-enriched regions, supporting an association between this motif and Wor1 binding. Motif similarity was interrogated using TOMTOM (version 4.9.1)[91].

### ChIP of H3K56Ac

White cells (CAY6678 and CAY6679) were taken from Spider plates and grown at 30°C overnight in the presence or absence of 5 mM nicotinamide. After 24 h, chromatin immunoprecipitation was performed as described previously [86] using an anti-H3K56Ac antibody (clone EPR996Y, Abcam, Cambridge, UK). Quantitative PCR was performed using primers listed in S10 Table and compared to input controls for each sample with three biological replicates.

### Statistical analysis

Statistics were performed using R (R Development Core Team) or Microsoft Excel (Microsoft Corporation). Statistical tests were performed as two-tailed Student’s t-test unless otherwise noted.

## Supporting Information

S1 Fig Comparative analysis of the shapes of white, hybrid, and opaque cells. The average eccentricity, form factor, and the ratio of maximum and minimum diameters were calculated for a population of white, hybrid and opaque cells. Error bars indicate standard error with 8 replicates.
(TIF)

S2 Fig Gene expression profiling of *C*. *tropicalis* cells in white, hybrid, and opaque states. (**A**) qRT-PCR measured abundance of candidate *C*. *tropicalis* white, hybrid, opaque, and state-specific genes. Abundance was measured for cells in logarithmic growth at 30°C and normalized to *CTRG_04189*, which was not differentially regulated in RNA-Seq data. (**B**) *C*. *tropicalis* gene expression levels are plotted; the x-axis shows relative expression counts and the y-axis is relative gene expression between white and opaque cells. *C*. *albicans* white-opaque regulated genes (from Hernday *et al*. [41]) with clear orthologs in *C*. *tropicalis* are highlighted as red (induced in *C*. *albicans* white cells) or blue (induced in *C*. *albicans* opaque cells). In general, the red and blue dots fail to cluster with being white-opaque regulated in *C*. *tropicalis*.
(TIF)

S3 Fig Switching behavior in mutant *C*. *tropicalis* strains. Analysis of switching mutants of *EFH1*, *CZF1*, *WOR2*, and *WOR3* compared to wildtype.
(TIF)

S4 Fig Analysis of *C*. *tropicalis* cells expressing transcription factors under the control of the regulatable *MAL2* promoter. White colonies (**A**) or opaque colonies (**B**) on non-inducing medium (-Maltose) and inducing medium (+Maltose) after growth at 30°C for 7 days. Cells from inducing medium (+Maltose) are shown. Phenotypes are indicated by “o” (opaque), “io” (invasive opaque), “o/h” (opaque/hybrid), “h” (hybrid), “w” (white), “sw” (smooth white), “fw” (filamentous white) or “sO” (smooth opaque). Scale bars = 5 μm.
(TIF)

S5 Fig Analysis of constitutive expression of *C*. *tropicalis* transcription factors on the white-opaque switch. Colony morphology (left) and cell morphology (right) from white parental cells (**A**) or opaque parental cells (**B**) transformed with the indicated transcription factor and grown on Spider medium at 30°C for 7 days. Phenotypes are indicated by “o” (opaque), “fo” (filamentous opaque), “h/o” (hybrid/opaque), “h” (hybrid), “w” (white), “sw” (smooth white), “fw” (filamentous white) or “so” (smooth opaque). Scale bars = 5 μm.
(TIF)

S6 Fig Analysis of *C*. *tropicalis* Wor1 DNA binding events at genes encoding white-opaque regulatory transcription factors. Binding of Wor1 was mapped by ChIP-Seq along the genomic loci of established or putative white-opaque transcriptional regulators. Positions of significant Wor1 binding are represented by red underlined regions.
(TIF)

S7 Fig *pMAL2*-induced cell states reflect *EFG1* and *WOR1* expression states. Total *EFG1* and *WOR1* expression levels were assayed by qRT-PCR in *C*. *tropicalis* white, hybrid, and opaque control cells, as well as in white and hybrid cells expressing the *pMAL2*-*WOR1* construct. For each strain, total *EFG1* and *WOR1* transcript levels were determined in medium both with and without maltose (+/- MAL, respectively). Error bars are standard deviations from three replicate experiments.
(TIF)

S8 Fig Analysis of the stability of induced phenotypic states. Stability of phenotypic states was analyzed in *C*. *tropicalis* cells that were originally in the white (**A**,**B**) or opaque (**C**,**D**) state. Cells were grown on inducing medium (Spider+Maltose) at 30°C for 7 days, and then transferred to non-inducing medium (Spider-Maltose) and grown for a further 7 days at 30°C to determine if cell states were maintained. Comparisons are between growth on inducing and non-inducing conditions, ** indicates p < 0.01 (Student’s t-test). (**B** and **D**) Colony and cell morphologies when cultured on Spider+Maltose (inducing) medium or when moved from inducing to Spider-Maltose (non-inducing) medium. Cell phenotypes are indicated by “w” (white), “h” (hybrid), “o” (opaque), and “fo” (filamentous opaque).
(TIF)

S9 Fig Effect of NAM and *pMAL2-WOR1* expression on white and hybrid cell phenotypes. (**A**) White cells were grown on Spider medium containing either 0, 5 mM, or 12.5 mM NAM for 7 days at 30°C and analyzed for colony and cellular phenotypes (+NAM). Cells from the induced hybrid (or control white) state were then grown for 7 days at 30°C in the absence of NAM and analyzed for colony and cellular phenotypes to assess heritability of the induced state (-NAM). (**B**) White or hybrid cells were grown in the presence of 5 mM NAM for 7 days at 30°C and analyzed for cellular phenotypes. Images show that cells had switched to hybrid and opaque states, respectively. However, these states were not stably maintained if re-cultured on medium without NAM (see Fig 6). (**C**) Cell images from colonies that stably inherited the induced state. White cells (top panel) or hybrid cells (bottom panel) were induced to switch by ectopic expression of *pMAL2-WOR1* and exposure to 5 mM NAM, resulting in conversion to hybrid and opaque states, respectively. These cells were then passaged twice for 7 days at 30°C on non-inducing medium (lacking both maltose and NAM), and cells imaged. Cells are shown to have stably maintained the induced state even after passaging.
(TIF)

S1 Table Analysis of single cells isolated from different phenotypic states. Single cells were picked from the indicated colonies using a micromanipulator and allowed to develop on Spider plates for 7 days at 30°C. A range of cell shapes was chosen to account for variable phenotypes from each state. In each case, 100% of the new colonies exhibited the phenotype of the original colony from which cells were picked. “% Sectors” indicates the percentage of colonies that contained minority sectors to alternative phenotypes as noted in parentheses.
(TIF)

S2 Table Global comparison of gene expression in white, opaque, and hybrid states. Log_2_ fold change refers to the difference in the average of the two cell states for each worksheet. ‘Gene’ refers to the common name and ‘Locus’ defines the contig position of the corresponding ORF. Test statistics refer to the delta method to estimate the distribution of the Jensen-Shannon metric as determined for p-values derived by CuffDiff analysis of transcript abundance differences and q-values are corrected for multiple hypothesis testing.
(XLSX)

S3 Table White, opaque, hybrid, and state-specific gene lists. State-specific genes are those that are differentially expressed between the three phenotypic states based on calculated q-values from S2 Table. White-specific genes were more numerous compared to the hybrid, opaque, and stage-specific sets because of the greater similarity in gene expression between the hybrid and opaque states. ‘Gene’ refers to the common name.
(XLSX)

S4 Table GO analysis of RNA-Seq data. GO term analysis was performed using the built in tool at the Candida Genome Database with corrections for multiple hypothesis testing. **‘**State-specific’ refers to Gene Ontology terms associated with genes that are differentially expressed between white, hybrid and opaque states. Significantly enriched GO terms were found for the white, opaque, and stage-specific categories whereas the hybrid state produced no significant associations due to a low number of genes with hybrid-specific expression.
(XLSX)

S5 Table Log_2_ FPKM values for white-opaque transcriptional regulators in *C*. *tropicalis*. (TIF)

S6 Table Log_2_ FPKM values for white-opaque transcriptional regulators in *C*. *albicans*. (TIF)

S7 Table Genomic regions enriched for Wor1 binding in *C*. *tropicalis* opaque cells. Regions that are significantly enriched for Wor1 binding from ChIP-Seq data are shown. Significantly enriched regions were identified using MACS2 and tested for reproducibility using IDR. The most proximal genes to each bound region are listed adjacent to the peak region. In cases where two genes reside immediately adjacent to the bound region in a head-to-head orientation, both genes are given.
(XLSX)

S8 Table Strains used in this study. (XLSX)

S9 Table Plasmids used in this study. (XLSX)

S10 Table Oligonucleotide table. (XLSX)

S11 Table Information and links to RNA-seq data. (XLSX)
