# Supplementary figures and images for: A Multistate Toggle Switch Defines Fungal Cell Fates and Is Regulated by Synergistic Genetic Cues
Source: PLoS Genet. 2016 Oct 6;12(10):e1006353. doi: 10.1371/journal.pgen.1006353 (PMC5053522; doi:10.1371/journal.pgen.1006353)

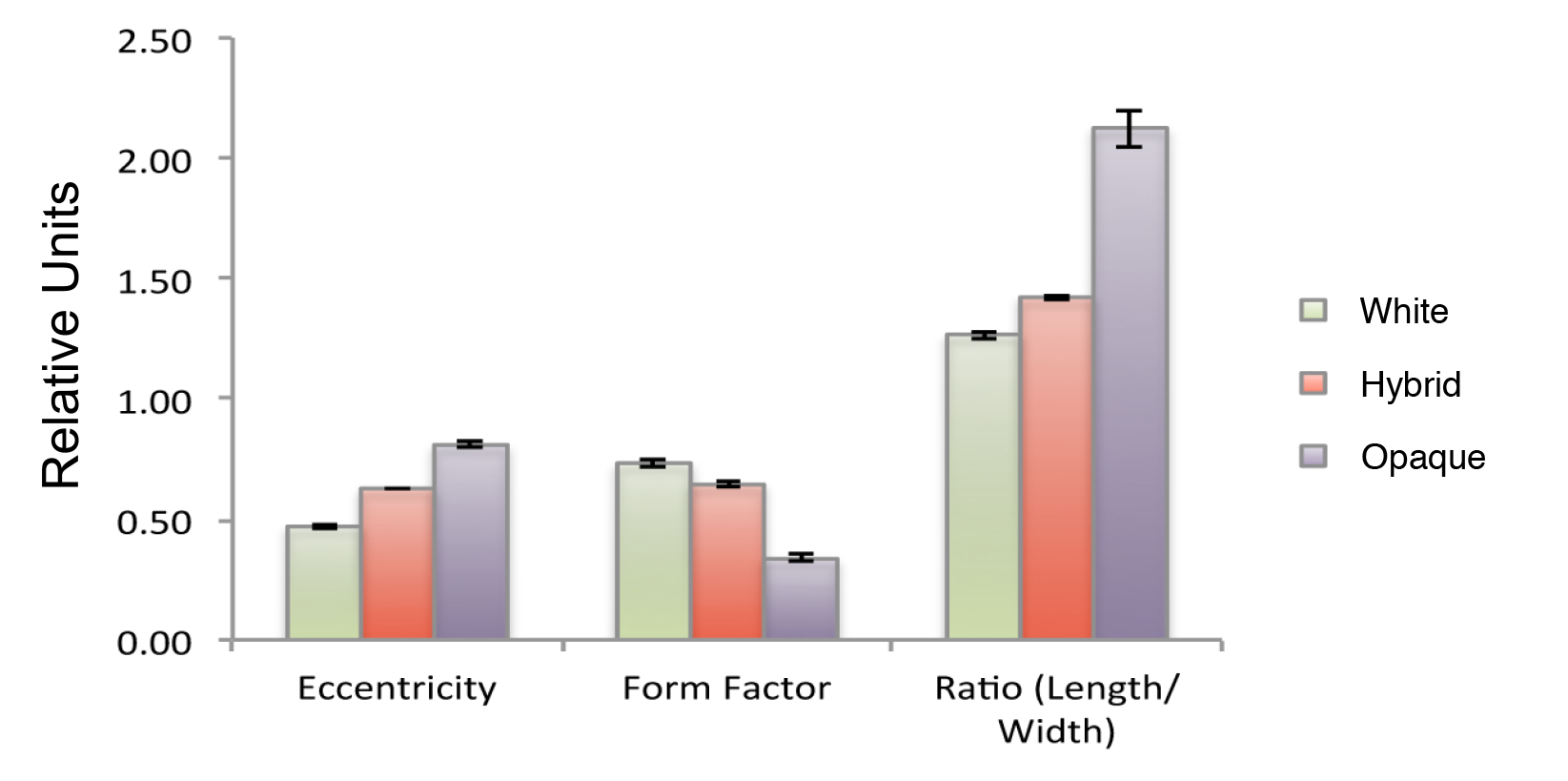

Supplement: S1 Fig — The average eccentricity, form factor, and the ratio of maximum and minimum diameters were calculated for a population of white, hybrid and opaque cells. Error bars indicate standard error with 8 replicates. (TIF) [file pgen.1006353.s001.tif]

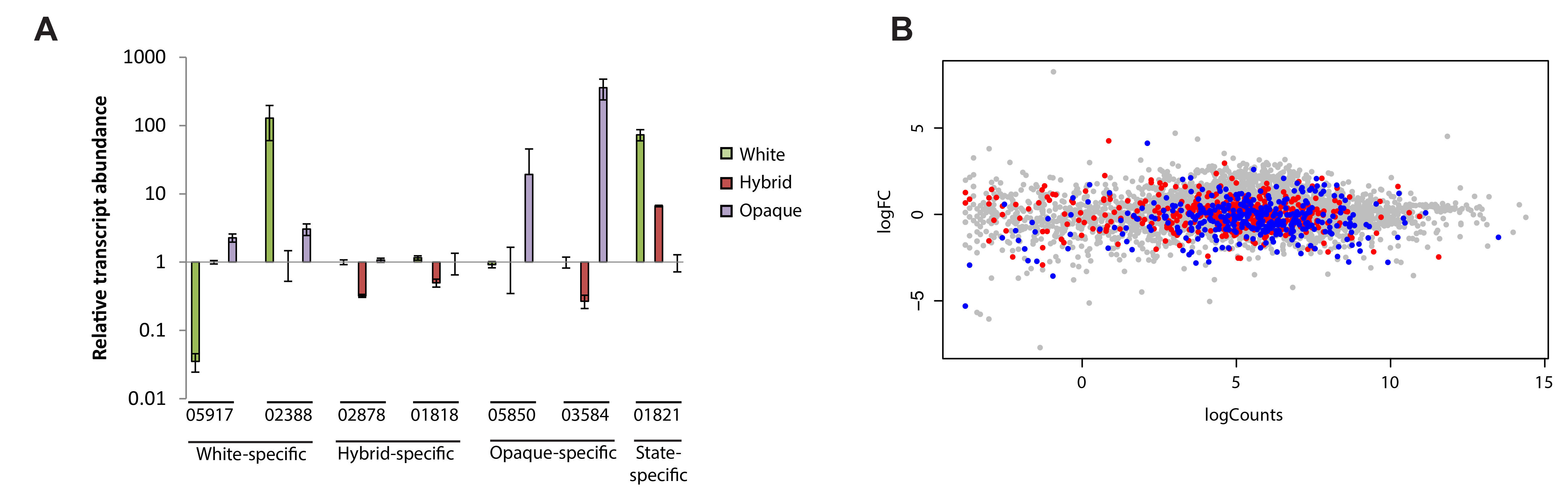

Supplement: S2 Fig — (A) qRT-PCR measured abundance of candidate C. tropicalis white, hybrid, opaque, and state-specific genes. Abundance was measured for cells in logarithmic growth at 30°C and normalized to CTRG_04189, which was not differentially regulated in RNA-Seq data. (B) C. tropicalis gene expression levels are plotted; the x-axis shows relative expression counts and the y-axis is relative gene expression between white and opaque cells. C. albicans white-opaque regulated genes (from Hernday et al. [41]) with clear orthologs in C. tropicalis are highlighted as red (induced in C. albicans white cells) or blue (induced in C. albicans opaque cells). In general, the red and blue dots fail to cluster with being white-opaque regulated in C. tropicalis. (TIF) [file pgen.1006353.s002.tif]

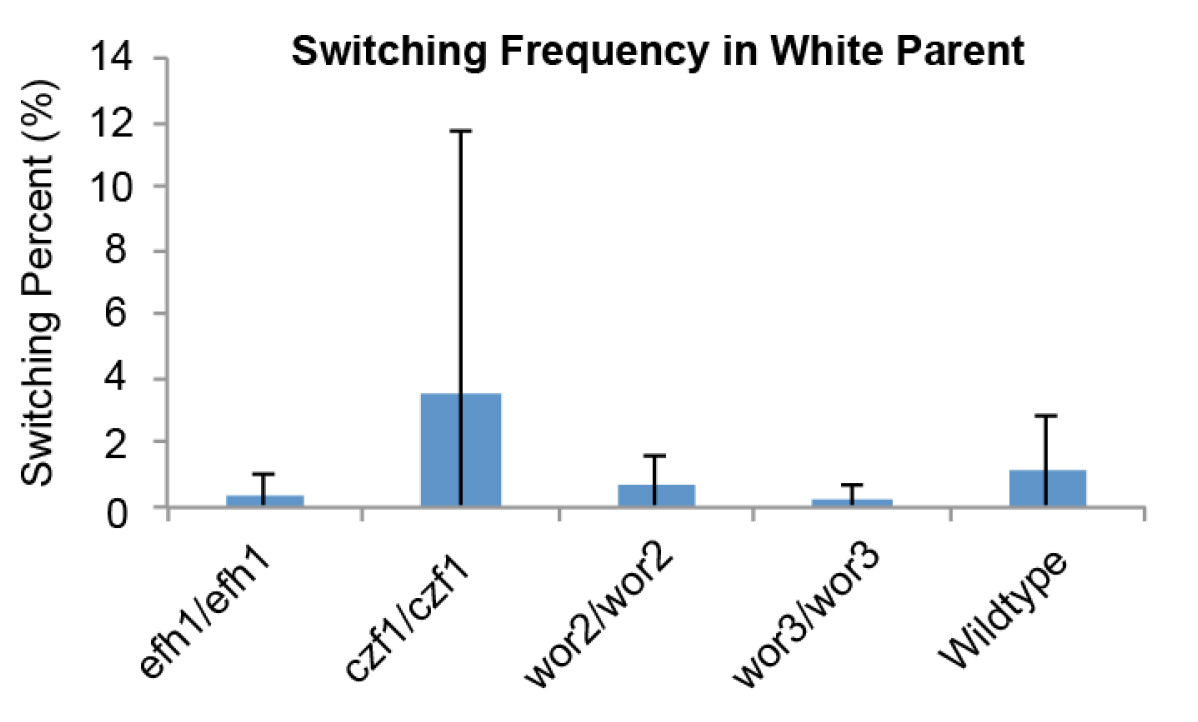

Supplement: S3 Fig — Analysis of switching mutants of EFH1, CZF1, WOR2, and WOR3 compared to wildtype. (TIF) [file pgen.1006353.s003.tif]

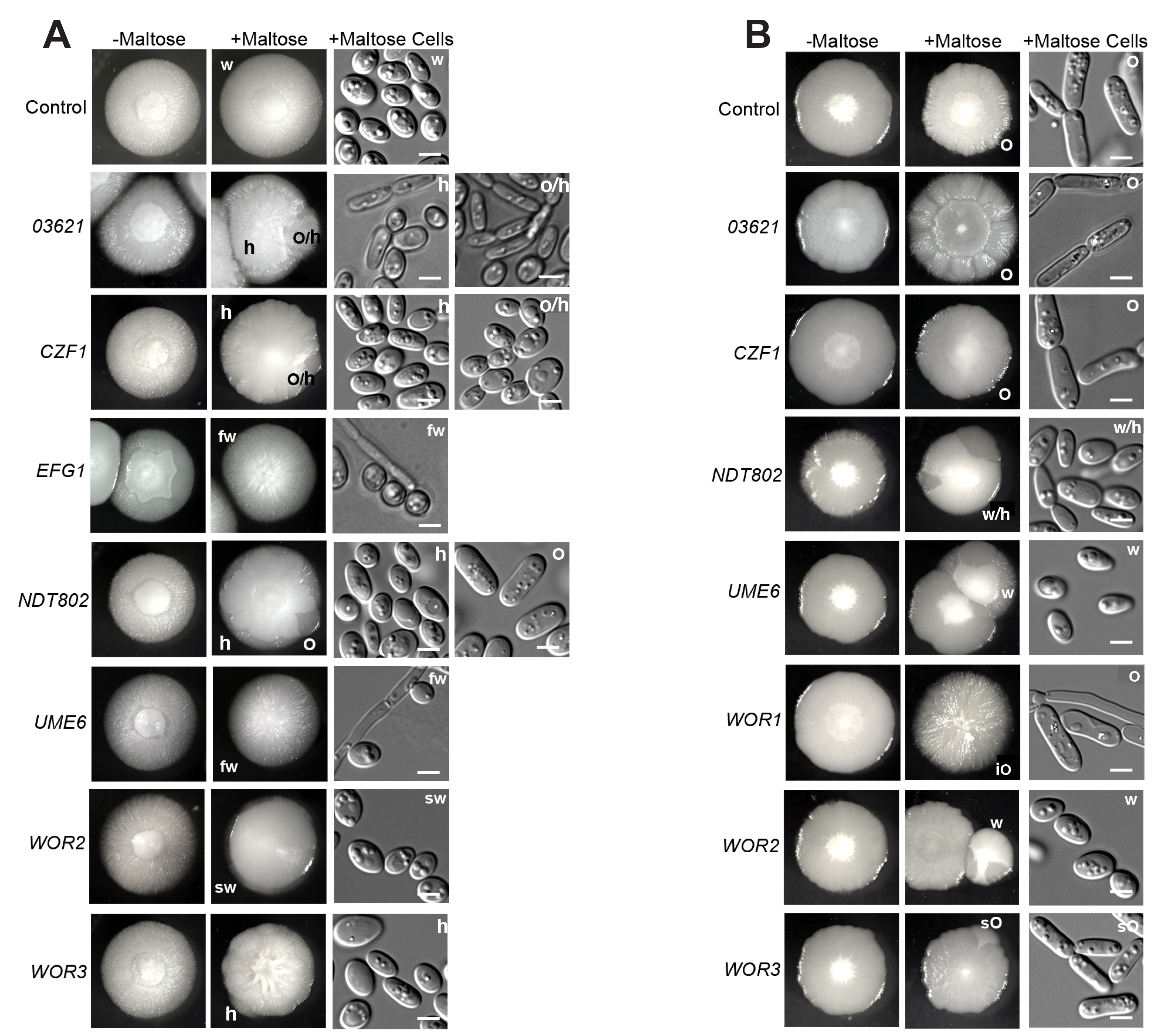

Supplement: S4 Fig — White colonies (A) or opaque colonies (B) on non-inducing medium (-Maltose) and inducing medium (+Maltose) after growth at 30°C for 7 days. Cells from inducing medium (+Maltose) are shown. Phenotypes are indicated by “o” (opaque), “io” (invasive opaque), “o/h” (opaque/hybrid), “h” (hybrid), “w” (white), “sw” (smooth white), “fw” (filamentous white) or “sO” (smooth opaque). Scale bars = 5 μm. (TIF) [file pgen.1006353.s004.tif]

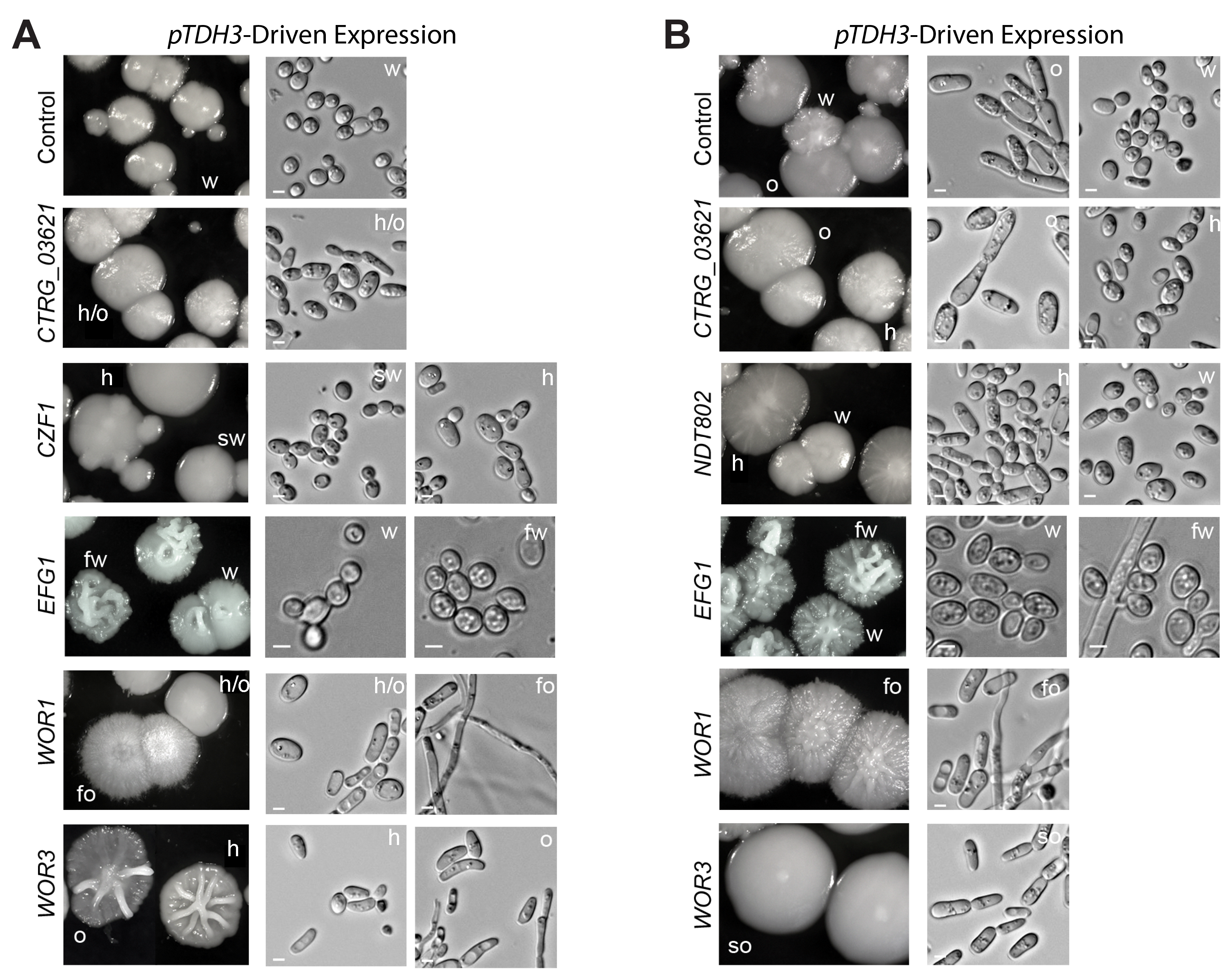

Supplement: S5 Fig — Colony morphology (left) and cell morphology (right) from white parental cells (A) or opaque parental cells (B) transformed with the indicated transcription factor and grown on Spider medium at 30°C for 7 days. Phenotypes are indicated by “o” (opaque), “fo” (filamentous opaque), “h/o” (hybrid/opaque), “h” (hybrid), “w” (white), “sw” (smooth white), “fw” (filamentous white) or “so” (smooth opaque). Scale bars = 5 μm. (TIF) [file pgen.1006353.s005.tif]

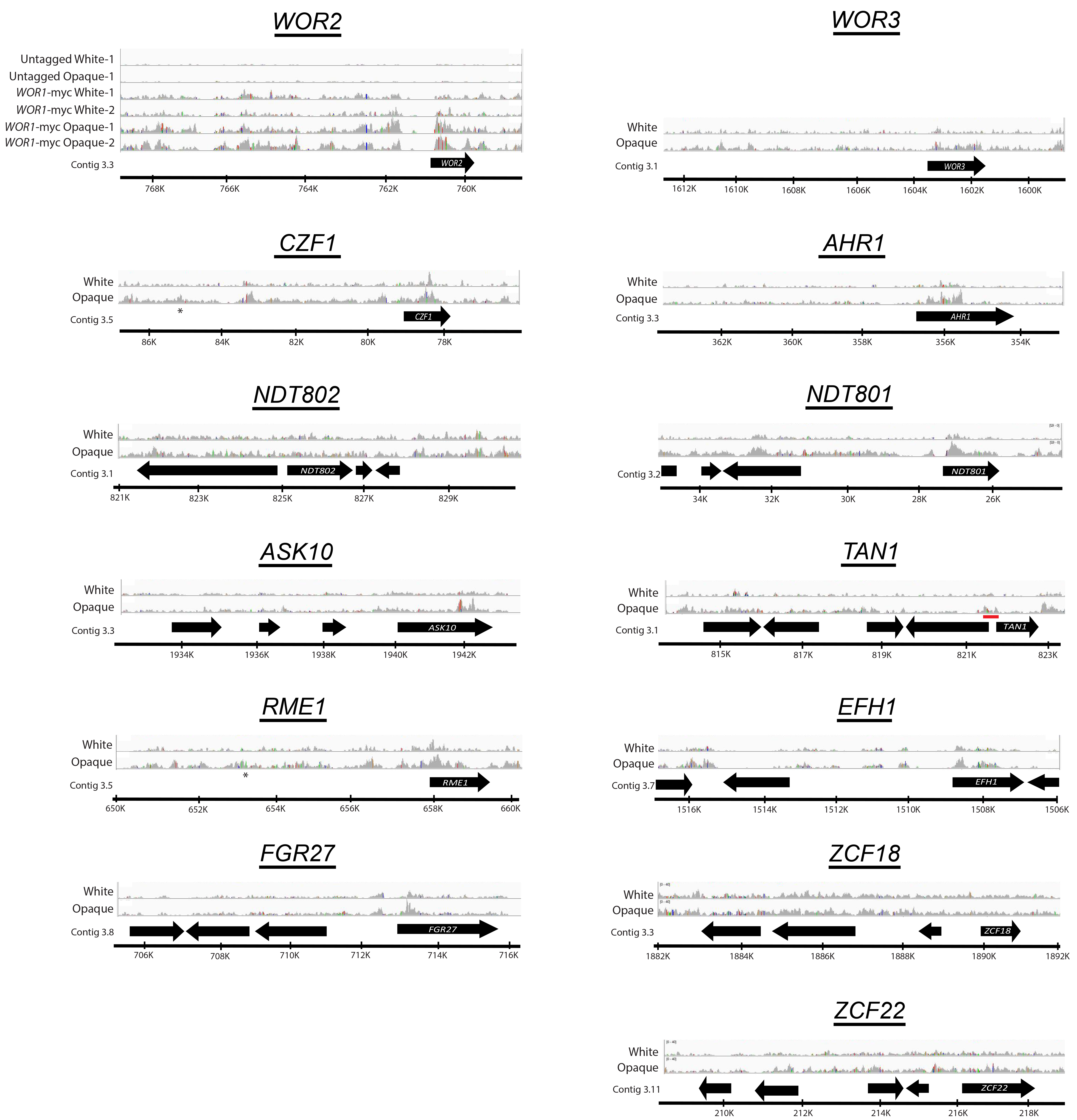

Supplement: S6 Fig — Binding of Wor1 was mapped by ChIP-Seq along the genomic loci of established or putative white-opaque transcriptional regulators. Positions of significant Wor1 binding are represented by red underlined regions. (TIF) [file pgen.1006353.s006.tif]

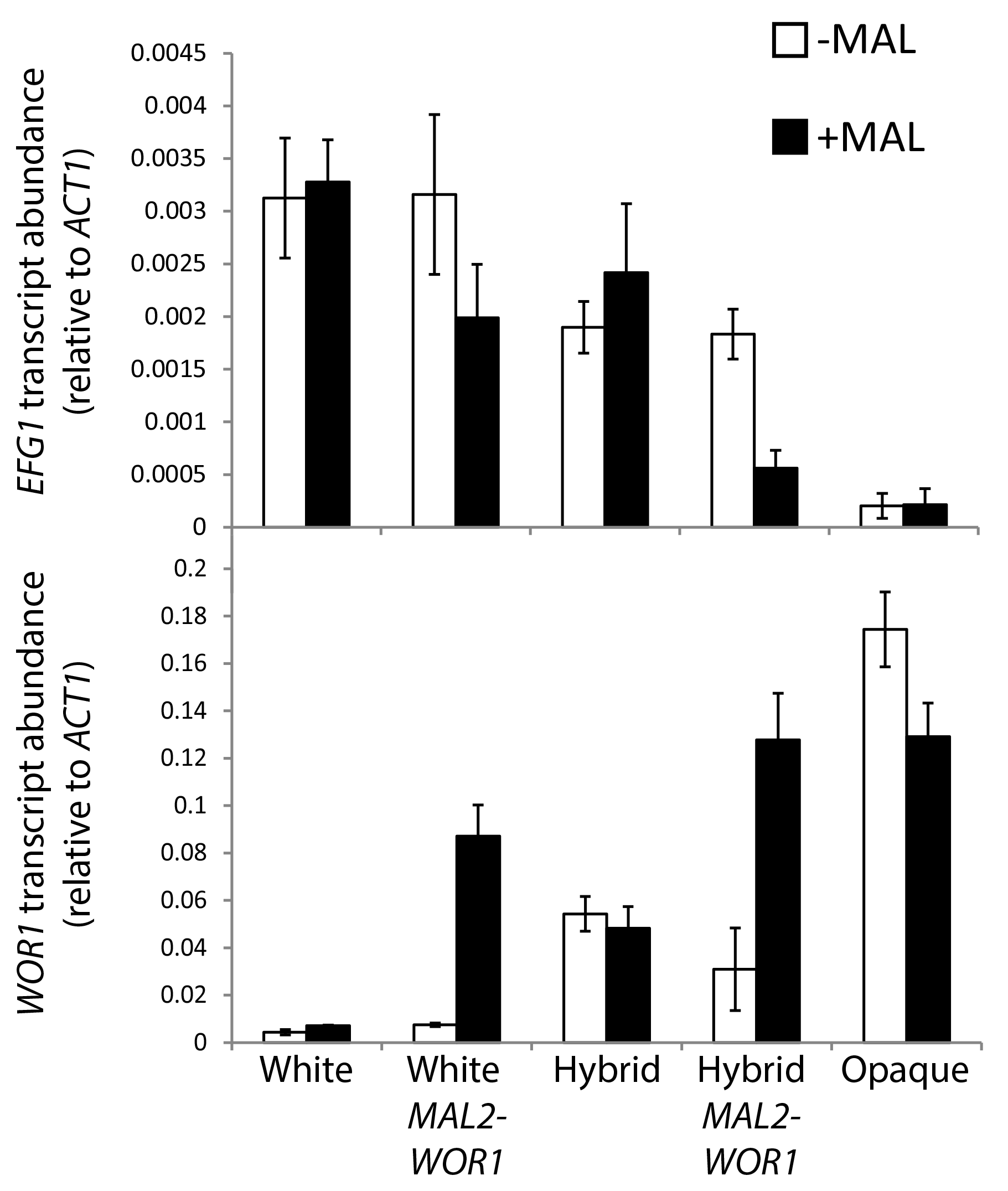

Supplement: S7 Fig — Total EFG1 and WOR1 expression levels were assayed by qRT-PCR in C. tropicalis white, hybrid, and opaque control cells, as well as in white and hybrid cells expressing the pMAL2-WOR1 construct. For each strain, total EFG1 and WOR1 transcript levels were determined in medium both with and without maltose (+/- MAL, respectively). Error bars are standard deviations from three replicate experiments. (TIF) [file pgen.1006353.s007.tif]

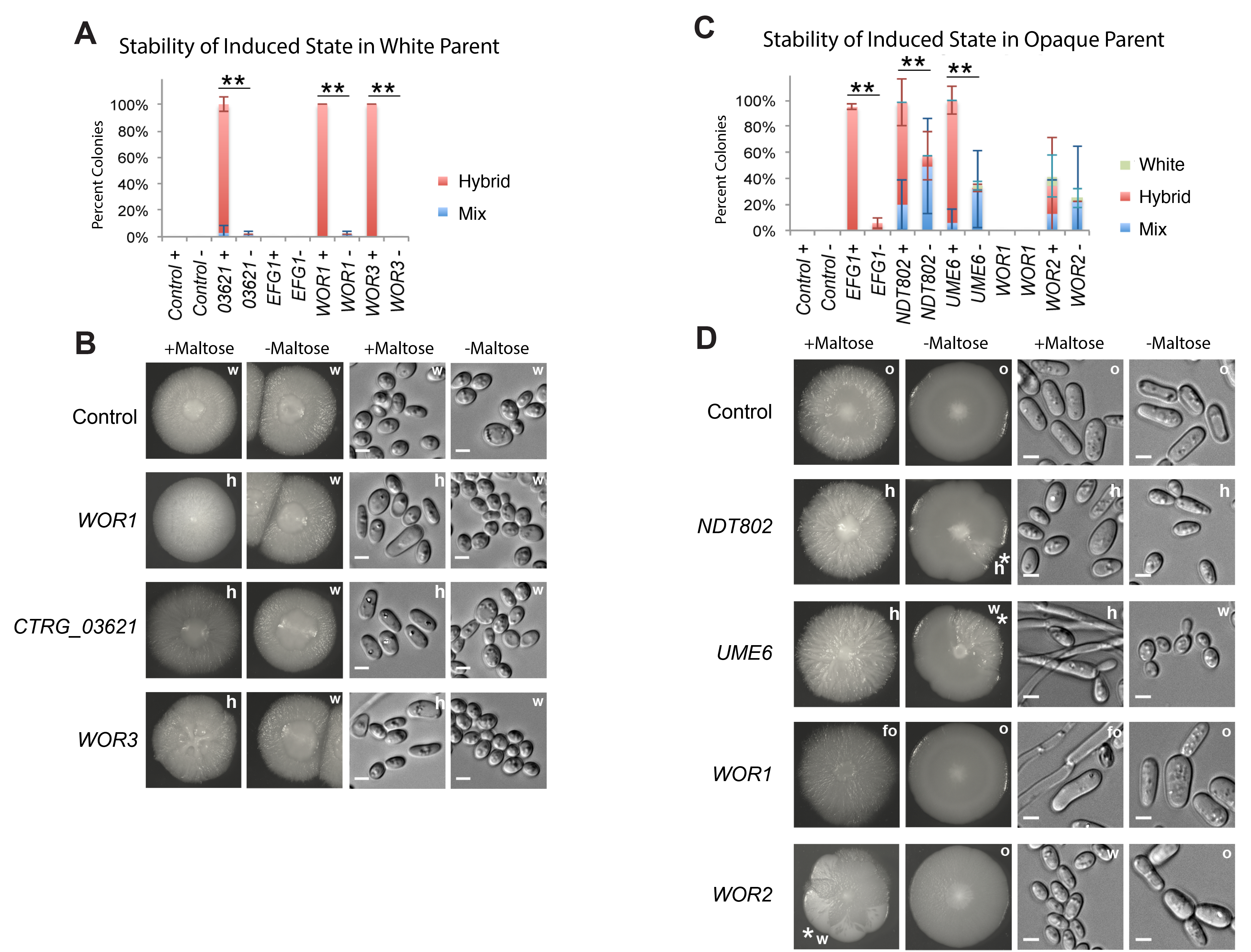

Supplement: S8 Fig — Stability of phenotypic states was analyzed in C. tropicalis cells that were originally in the white (A,B) or opaque (C,D) state. Cells were grown on inducing medium (Spider+Maltose) at 30°C for 7 days, and then transferred to non-inducing medium (Spider-Maltose) and grown for a further 7 days at 30°C to determine if cell states were maintained. Comparisons are between growth on inducing and non-inducing conditions, ** indicates p < 0.01 (Student’s t-test). (B and D) Colony and cell morphologies when cultured on Spider+Maltose (inducing) medium or when moved from inducing to Spider-Maltose (non-inducing) medium. Cell phenotypes are indicated by “w” (white), “h” (hybrid), “o” (opaque), and “fo” (filamentous opaque). (TIF) [file pgen.1006353.s008.tif]

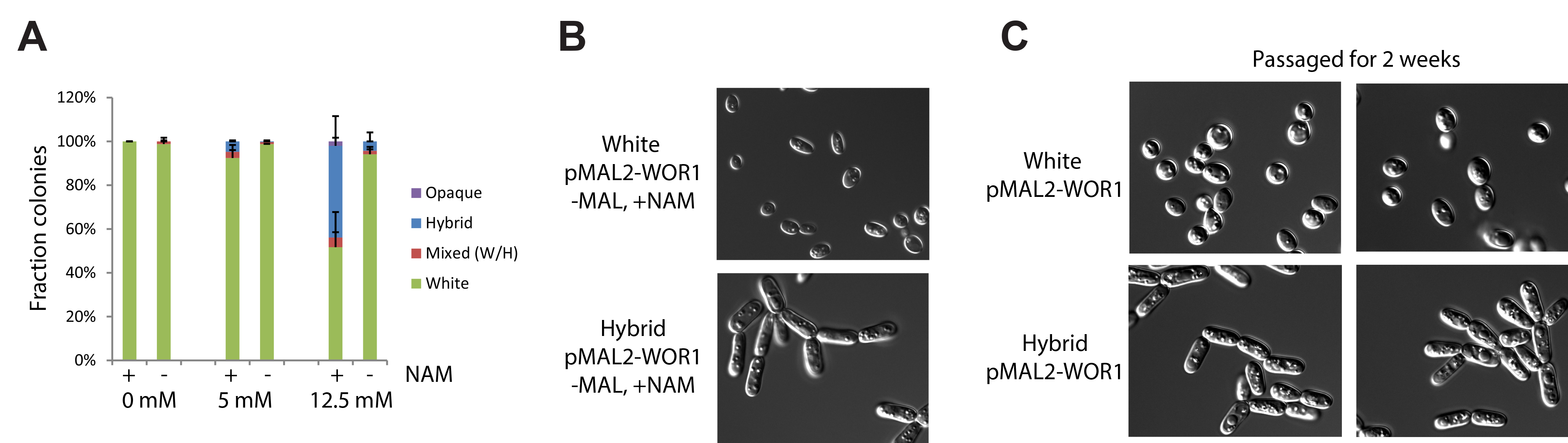

Supplement: S9 Fig — (A) White cells were grown on Spider medium containing either 0, 5 mM, or 12.5 mM NAM for 7 days at 30°C and analyzed for colony and cellular phenotypes (+NAM). Cells from the induced hybrid (or control white) state were then grown for 7 days at 30°C in the absence of NAM and analyzed for colony and cellular phenotypes to assess heritability of the induced state (-NAM). (B) White or hybrid cells were grown in the presence of 5 mM NAM for 7 days at 30°C and analyzed for cellular phenotypes. Images show that cells had switched to hybrid and opaque states, respectively. However, these states were not stably maintained if re-cultured on medium without NAM (see Fig 6). (C) Cell images from colonies that stably inherited the induced state. White cells (top panel) or hybrid cells (bottom panel) were induced to switch by ectopic expression of pMAL2-WOR1 and exposure to 5 mM NAM, resulting in conversion to hybrid and opaque states, respectively. These cells were then passaged twice for 7 days at 30°C on non-inducing medium (lacking both maltose and NAM), and cells imaged. Cells are shown to have stably maintained the induced state even after passaging. (TIF) [file pgen.1006353.s009.tif]

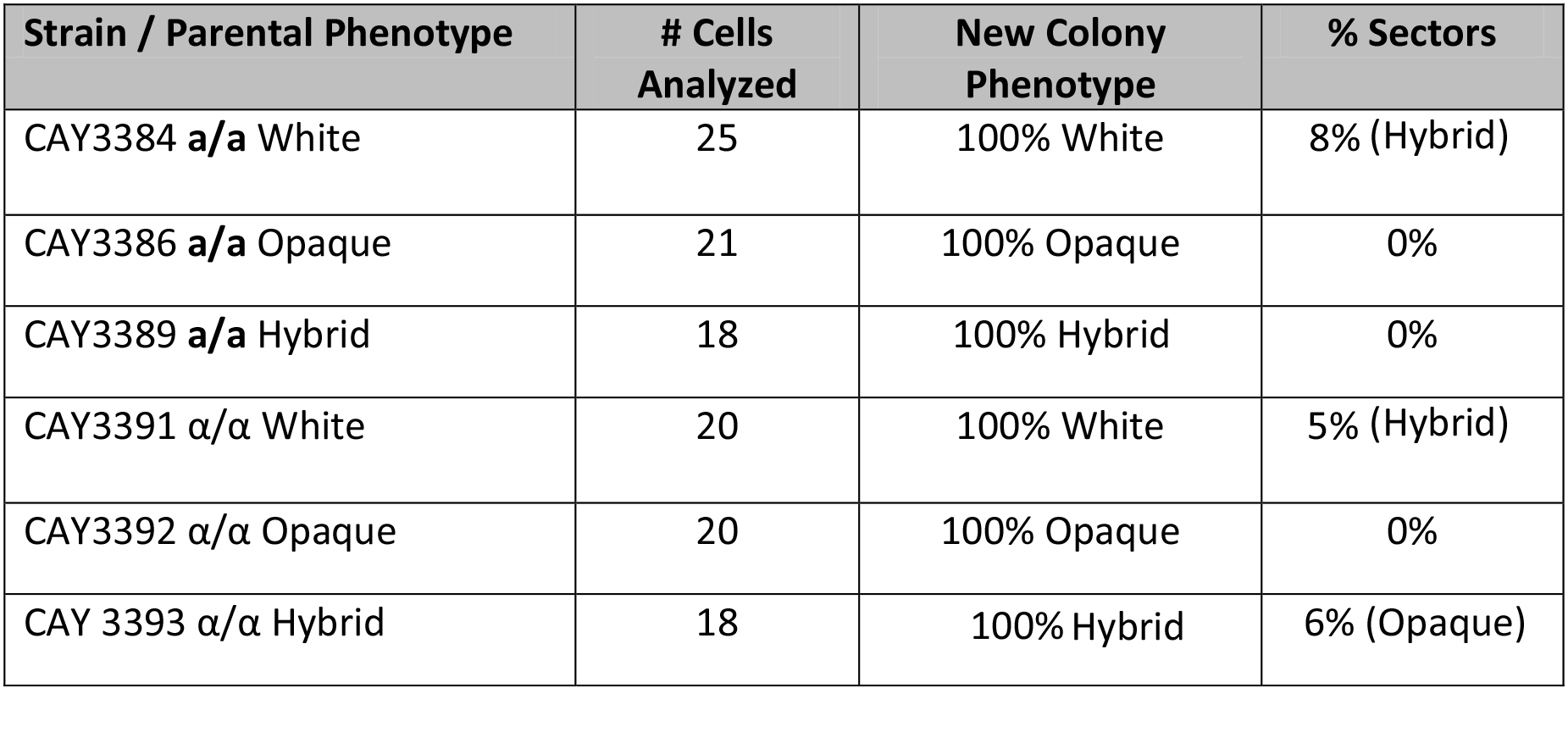

Supplement: S1 Table — Single cells were picked from the indicated colonies using a micromanipulator and allowed to develop on Spider plates for 7 days at 30°C. A range of cell shapes was chosen to account for variable phenotypes from each state. In each case, 100% of the new colonies exhibited the phenotype of the original colony from which cells were picked. “% Sectors” indicates the percentage of colonies that contained minority sectors to alternative phenotypes as noted in parentheses. (TIF) [file pgen.1006353.s010.tif]

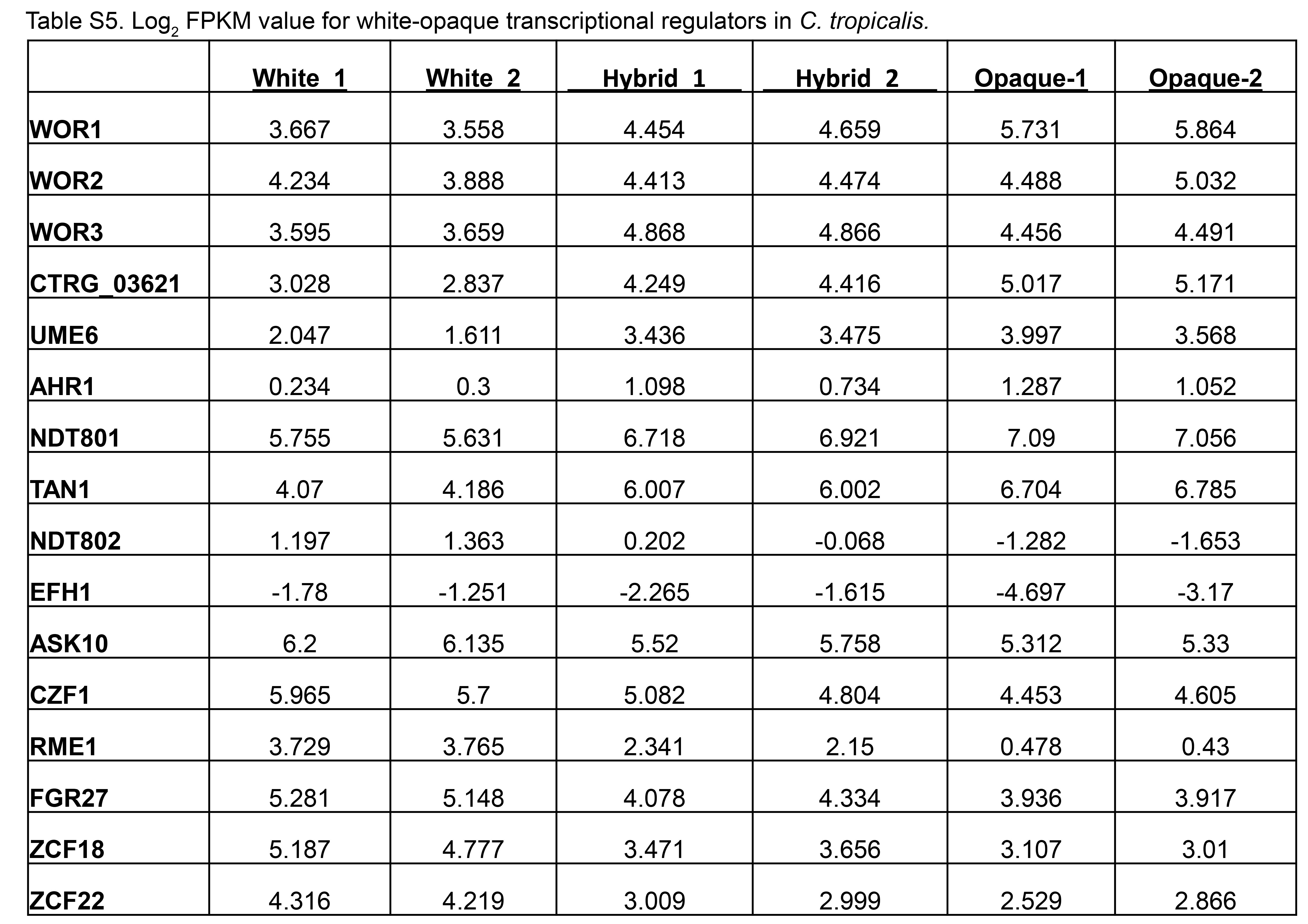

Supplement: S5 Table — (TIF) [file pgen.1006353.s014.tif]

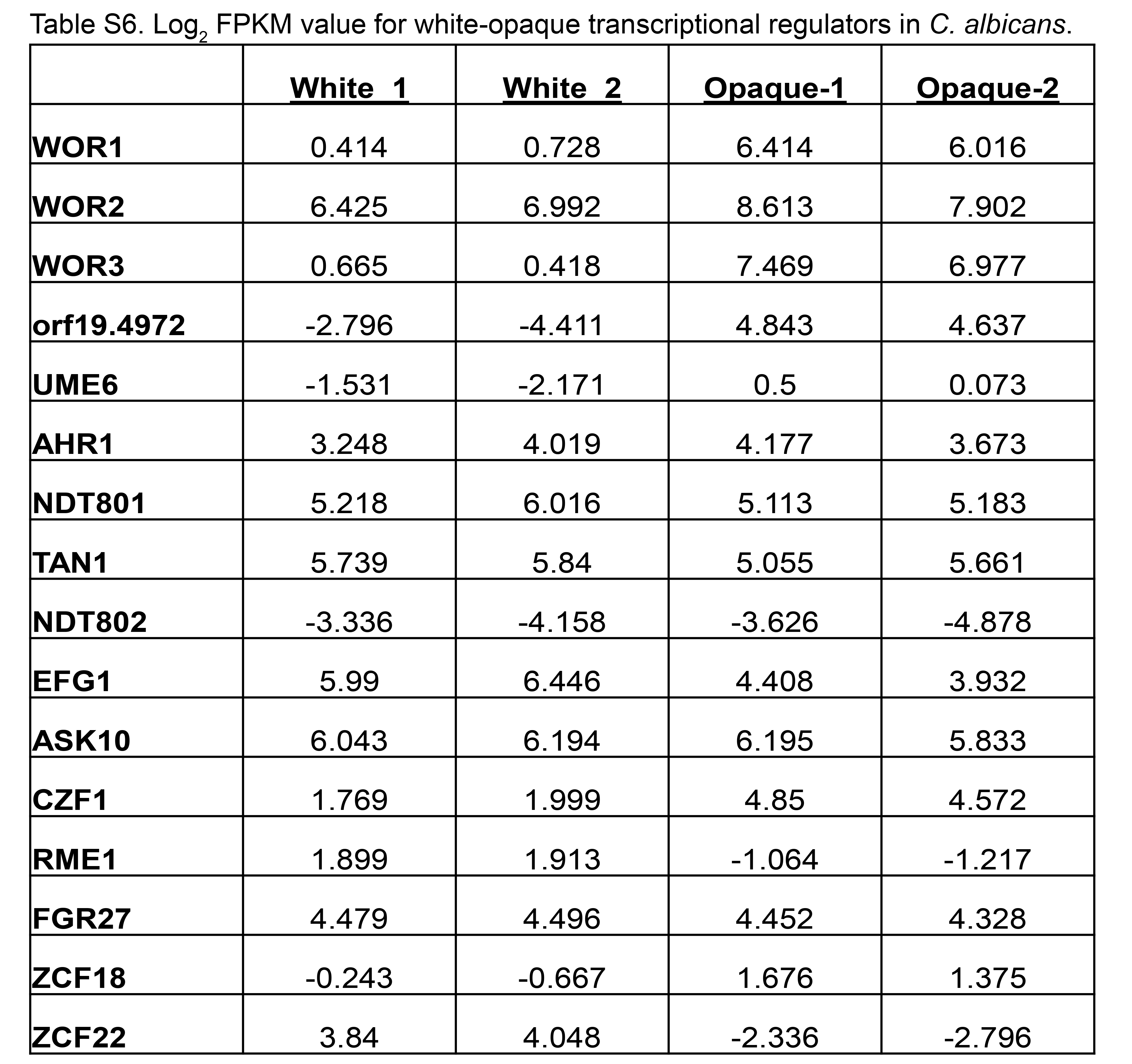

Supplement: S6 Table — (TIF) [file pgen.1006353.s015.tif]
